# Plasmonic Hot‐Carrier Redox Enables Proton‐Coupled Electron Transfer at C─H Bonds

**DOI:** 10.1002/anie.202518818

**Published:** 2025-12-17

**Authors:** Daniel Velev Latchev, Arthur Andreis, Julian Michael Heeg, Jacinto Sá

**Affiliations:** ^1^ Department of Chemistry‐Ångström Uppsala University Uppsala 751 20 Sweden; ^2^ Institute of Physical Chemistry Polish Academy of Sciences Warsaw 01–224 Poland

**Keywords:** C─H activation, Mechanistic study, PCET, Photoredox catalysis, Plasmonics

## Abstract

Proton‐coupled electron transfer (PCET) enables the activation of strong X─H bonds (X = C, N, O) under mild conditions, yet mechanistic interrogation remains challenging due to limited control over electron and proton energetics. Here, we demonstrate that plasmon‐driven photocatalysis provides a powerful platform to disentangle these contributions. Using gold nanoparticles supported on an engineered energy‐filter substrate as photoelectrodes, we achieve oxidative C─H bond activation in 1‐benzyl‐1,4‐dihydronicotinamide via multi‐site PCET under visible‐light excitation. Photocurrent kinetics and energetics zone analysis reveal an electron‐then‐proton transfer (ET–PT) pathway that requires pre‐association of the base. The average plasmonic oxidation potential is estimated at ∼0.64 V versus Fc⁺/Fc, corresponding to hot‐hole energies of ∼0.52 eV. These findings establish plasmonic materials not only as versatile light absorbers for photoredox catalysis but also as mechanistic probes for resolving fundamental PCET pathways.

## Graphical abstract

Plasmonic hot carriers provide unprecedented control over coupled electron–proton transfer, enabling visible‐light activation of strong C─H bonds. Using an energy‐filter electrode, we reveal a stepwise electron‐then‐proton pathway and directly tune the underlying driving forces. This work positions plasmonic nanostructures as a powerful new platform for uncovering fundamental reaction mechanisms.

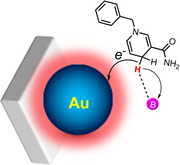

Proton‐coupled electron transfer (PCET) underlies many of nature's most demanding redox processes and has emerged as a central principle in synthetic catalysis, enabling the activation of strong and otherwise inert X─H bonds (X = N, O, C) under mild conditions.^[^ ^1^, ^2^, ^3^ ^]^ By coupling electron and proton motion, PCET lowers activation barriers and avoids high‐energy ionic intermediates, thereby imparting control and selectivity in transformations ranging from enzymatic reactions to photoredox catalysis.^[^ ^4^ ^]^

A particularly powerful variant is multi‐site PCET (MS‐PCET), in which the electron and proton are transferred to distinct acceptors. This spatial separation decouples redox potential from acidity, allowing the effective bond dissociation free energy (“BDFE”)^[^ ^3^ ^]^ to be independently tuned. The “BDFE” represents the thermodynamic driving force for concerted and stepwise proton–electron exchange and is determined by the combined reduction potential of the electron acceptor and the p*K*_a_ of the proton acceptor. As a result, MS‐PCET can access reactivity regimes beyond the reach of unimolecular hydrogen atom transfer reagents, enabling activation of particularly strong X─H bonds under mild, controlled conditions.^[^ ^5^ ^]^

This tunability of the “BDFE” through MS‐PCET has been leveraged to broaden the chemical scope of PCET reactivity. Introduced into photoredox catalysis by the Knowles group, MS‐PCET has enabled selective C─H,^[^ ^6^, ^7^ ^]^ N─H^[^ ^8^ ^]^ and O─H^[^ ^9^ ^]^ activations under mild conditions, granting access to highly reactive intermediates that are otherwise inaccessible. By separating electron and proton transfer pathways, MS‐PCET also enhances chemoselectivity, as only substrates that simultaneously satisfy both redox and acid–base driving forces undergo activation. This conceptual flexibility establishes MS‐PCET as a powerful design principle for selective transformations and motivates ongoing efforts to elucidate its mechanistic foundations.

Despite this promise, current experimental approaches face significant limitations. In dye‐sensitized photoredox systems, the most widely used platform, the light‐absorber absorption profile, redox potential, and excited‐state lifetime are intrinsically linked, making it difficult to independently tune electron‐driving forces.^[^ ^10^, ^11^ ^]^ Electrocatalytic methods, by contrast, offer precise control over potential^[^ ^12^ ^]^ but lack spatial selectivity and efficient coupling to proton transfer events, limiting their mechanistic insight. Consequently, it remains challenging to develop experimental platforms in which electron and proton energetics can be adjusted independently under catalytically relevant conditions.

Plasmonic nanostructures offer a fundamentally distinct strategy for light‐driven PCET reactivity. Unlike molecular dyes or semiconductors with fixed band gaps, metallic nanoparticles harvest visible light through collective plasmon oscillations, generating energetic “hot” carriers with a broadened Fermi–Dirac distribution.^[^ ^13^, ^14^, ^15^, ^16^ ^]^ This produces a continuum of accessible redox potentials within a single structure, enabling oxidation and reduction processes across a wide energetic window.^[^ ^16^, ^17^ ^]^ Moreover, the ultrafast decay of plasmons yields exceptionally high carrier fluxes and intense local electromagnetic fields at the metal–solution interface, which can markedly accelerate interfacial charge transfer to molecular substrates.^[^ ^18^, ^19^ ^]^ These features make plasmonic nanoparticles uniquely powerful platforms for probing PCET pathways and controlling charge‐transfer energetics under catalytically relevant conditions.

Here, we investigate the plasmon‐driven activation of 1‐benzyl‐1,4‐dihydronicotinamide (BNAH) as a model system for MS‐PCET at C─H bonds.^[^ ^20^, ^21^ ^]^ By coupling plasmonic hot‐carrier generation with base‐assisted proton transfer, we demonstrate that metallic nanoparticles can mediate otherwise challenging C─H bond oxidations under mild, visible‐light irradiation. This study establishes plasmonic photocatalysts as a versatile and tunable platform for controlling redox energetics in PCET and reveals their potential as mechanistic probes for understanding and advancing selective photoredox transformations.

The plasmonic electrode used in this study is based on an energy‐filter concept that we have reported previously,^[^ ^22^, ^23^ ^]^ and its operating mechanism is illustrated schematically in Figure S1 of the Supporting Information (SI). An energy filter enables the selective extraction of plasmon‐generated hot carriers by imposing an energetic threshold for charge transfer. When metallic nanoparticles absorb visible light, plasmon decay produces a broad distribution of excited electrons and holes. By interfacing the metal with a dielectric layer of defined band alignment, only carriers above a specific energy can traverse the interface and participate in interfacial reactions. This energetic gating effectively isolates high‐energy carriers from the thermalized Fermi population, allowing precise tuning of redox driving forces and providing mechanistic insight into plasmon‐induced charge transfer.

The details of electrode fabrication and characterization have been reported elsewhere.^[^ ^23^ ^]^ Briefly, a pinhole‐free amorphous TiO_2_ layer (∼10 nm thick) was deposited onto fluorine‐doped tin oxide (FTO) glass to serve as an ultrathin dielectric barrier between the collector (FTO) and the plasmonic nanostructure. This layer acts as an energetic filter, exhibiting a step‐like transmission profile^[^ ^24^ ^]^ that selectively allows hot electrons with energies above ∼0.5–0.6 eV (corresponding to the TiO_2_ work function of 4.4–4.5 eV) to transfer from the plasmonic particles,^[^ ^25^ ^]^ while blocking low‐energy electrons and all hot holes. Consequently, the filter enhances interfacial hot‐carrier separation and prolongs the lifetime and energetics of the hot holes, making them available for catalytic reactions. Gold nanoparticles (Au NPs) were then fabricated directly on the energy‐filter substrate via thermal annealing of thin Au films at 723 K for 30 min, producing a dense distribution of metallic spheres with diameters of 10–20 nm (average ∼15 nm), as confirmed by scanning electron microscopy (Figure S2). The resulting nanostructures exhibited a localized surface plasmon resonance band centered at ∼610 nm (Figure S3).^[^ ^23^ ^]^

Oxidation of nicotinamides, such as 1‐benzyl‐1,4‐dihydronicotinamide (BNAH), proceeds through a two‐electron process accompanied by the loss of one proton, yielding the corresponding pyridinium ion (BNA^+^).^[^ ^3^ ^]^ The radical–cation intermediates formed during this process are strongly acidic, whereas the closed‐shell reduced species are weak acids, underscoring their intrinsic tendency to engage in PCET rather than purely electron‐transfer (ET) or proton‐transfer (PT) pathways.^[^ ^20^, ^21^ ^]^ Previous mechanistic studies with molecular oxidants and bases have shown that stepwise electron‐first or proton‐first sequences are generally favored, while the fully concerted electron–proton transfer (CEPT) pathway is accessible only within narrow thermodynamic windows and is often limited by large donor–acceptor separations.^[^ ^26^ ^]^ It should be mentioned that the CEPT process, in which the proton and electron are transferred simultaneously, is synthetically preferred because it avoids the formation of high‐energy charged intermediates or transition states characteristic of stepwise mechanisms.

Figure 1a presents the square scheme illustrating the pathways leading to the formation of the neutral radical (BNA^•^). The subscript notation indicates the sequence of ET and PT steps. Formation of BNA^•^ is the rate‐limiting step, as the subsequent one‐electron oxidation to BNA⁺ (*E* ^○^ = −1.08 V versus SCE in acetonitrile^[^ ^20^ ^]^) is both thermodynamically and kinetically favored. Formation of BNA^•^ is the main factor underlying the photocurrent profiles discussed below, for the following reasons. First, the C─H bond dissociation free energy (BDFE) of BNAH in acetonitrile is 86.9 kcal·mol^−1^,^[^ ^3^ ^]^ which is markedly lower than the amide N─H BDFE, estimated to be around 107 kcal·mol^−1^.^[^ ^27^ ^]^ To substantiate this argument, reactions performed under identical conditions using acetamide showed no measurable photocurrent. Second, the anodic breakdown of acetonitrile occurs at 2.0–2.3 V versus SCE,^[^ ^28^ ^]^ making solvent oxidation with the current photoelectrode not likely. Third, photocurrent generation via plasmon energy‐transfer processes is highly unlikely under the tested conditions,^[^ ^29^, ^30^ ^]^ given the efficient extraction of hot electrons achieved through the energy‐filter concept,^[^ ^22^ ^]^ and on such processes there is a need for simultaneous transfer of electrons and holes. To further substantiate this, olefin [2 + 2] cycloaddition with styrene, a classical diagnostic reaction for energy‐transfer pathways,^[^ ^31^ ^]^ was carried out under the same conditions, but no photocurrent beyond the background was detected.

**Figure 1:**
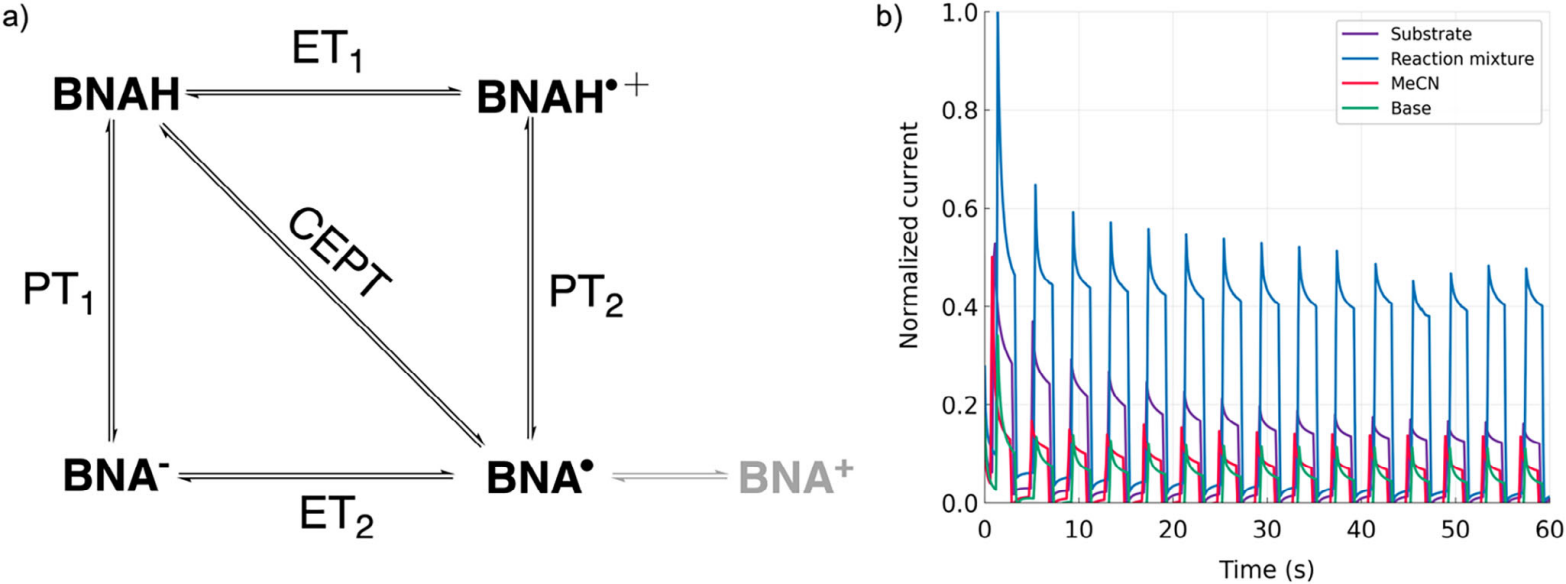
a) Square. scheme illustrating the PCET mechanisms of BNAH; b) Representative chronoamperometry traces used to extract kinetic information under 50% laser illumination. *Substrate* = reaction medium contained 2 mM BNAH, 100 mM TBAPF_6_ (supporting electrolyte) in acetonitrile; *Reaction mixture* = reaction medium contained 2 mM BNAH, 80 mM pyridine,100 mM TBAPF_6_ (supporting electrolyte) in acetonitrile; *MeCN* = 100 mM TBAPF_6_ (supporting electrolyte) in acetonitrile; and *Base* = reaction medium contained 80 mM pyridine, 100 mM TBAPF_6_ (supporting electrolyte) in acetonitrile.

Catalytic activity was quantified from photocurrent measurements in a two‐electrode configuration, using the plasmonic electrode as the working electrode and a Pt wire as the counter electrode, under pulsed‐light excitation. The illuminated electrode area was 0.95 cm^2^. A continuous‐wave laser at 635 nm (maximum power density: 130 mW·cm^−2^) was employed as the light source, with modulation at a repetition rate of 0.5 Hz. This repetition rate was found to be optimal for isolating photocatalytic contributions from thermal effects, which are commonly encountered in plasmonic research. The experiments were performed at two illumination intensities, corresponding to 50% (i.e., 65 mW·cm^−2^) and 60% (i.e., 52 mW·cm^−2^) attenuation of the maximum laser power, with the dual purpose of assessing the effect of light intensity and mitigating electrode photodegradation. Owing to the semi‐transparent nature of the photoelectrode, only ∼15% of the incident light was absorbed by the plasmonic particles, ensuring that the study remained well below the multi‐electron reaction regime.^[^ ^32^ ^]^ Further details are provided in the Supporting Information. Note that quantification of the isolated product is very challenging given the closed electrochemical setup employed. Under these conditions, the photoproduct is likely converted back to the original substrate, as this is the most probable reaction occurring at the counter electrode due to the high cathodic limit of the solvent (approximately ‐−3.0 V versus SCE).^[^ ^33^ ^]^ This was further supported by the absence of distinct product peaks in the proton NMR spectrum of the reaction mixture.

This study employed seven different nitrogen heterocyclic bases, whose identities and p*K*_a_ values are listed in Table 1.^[^ ^34^ ^]^ By selecting nearly isomorphic structures with the same proton‐accepting atom, we aimed to minimize the influence of structural differences, thereby isolating the effect of the bases’ p*K*_a_ on the study outcome. A substrate‐to‐base ratio of 1:40 was selected, as this ratio has previously been reported as optimal.^[^ ^26^ ^]^ To investigate electron‐transfer kinetics during photoelectrochemical operation, we employed our previously developed adaptation^[^ ^22^ ^]^ of the Chidsey formalism,^[^ ^35^ ^]^ which enables extraction of rate constants from transient current decays following a stimulus (in this case, a light pulse). Complementary Cottrell analysis for planar electrodes^[^ ^36^ ^]^ was used to identify the onset of diffusion‐limited photocurrent contributions. Together, these operando diagnostics, combined with controlled light modulation, provide direct insight into catalytic activity while minimizing heat accumulation at the electrode.^[^ ^22^ ^]^

**Table 1:** Identities and p*K*_a_ values (in acetonitrile) of the bases used in this study, along with the driving forces ($-\Delta G^{\circ })$ for proton transfer from BNA^•+^ and for PCET in the corresponding reaction mixtures.

Base	$pK_{a(H^{+}base)}\;\mathrm{in}\;\mathrm{MeCN}$ [28]	$-\Delta G_{PT}^{\circ }$ (eV)^a)^	− Δ*G* ^○^_*PCET*_ (eV)
2,6‐dimethoxypyridine	7.7	0.17	0.64
3‐chloropyridine	9.6	0.29	0.76
3‐acetylpyridine	10.8	0.36	0.83
Pyridine	12.5	0.46	0.93
4‐methoxypyridine	14.2	0.56	1.03
4‐aminopyridine	17.6	0.76	1.23
Piperidine	18.9	0.88	1.35

^a)^ $pK_{a({BNAH}^{\cdot +})}$ = 4.7^[^ ^3^ ^]^

Figure 1b shows a representative photocurrent response of a reaction mixture under 50% laser power attenuation. The signals are normalized to facilitate comparison across compositions. All experiments were conducted using the same electrode under identical illumination conditions, thereby eliminating these parameters as sources of variation in the observed responses. Complete datasets are provided in the Supporting Information: Figures S4–S10 (50% attenuation) and Figures S11–S17 (60% attenuation). Control measurements with only base in the electrolyte solution produced current values comparable to those of the electrolyte alone (MeCN sample), confirming that no oxidative process occurs and that the signal arises solely from background current. In contrast, the mixture containing only the substrate in electrolyte generated a measurable photocurrent, attributable to oxidation of BNAH to BNA^•+^, consistent with its redox potential (*E* = 0.57–0.79 V versus SCE).^[^ ^37^, ^38^ ^]^ Notably, introducing base into this mixture resulted in nearly a threefold increase in current, strongly suggesting that proton‐coupled electron transfer (PCET) plays a key role in enhancing catalytic output. Notably, when the electrode lacked Au NPs, the photocurrent was entirely undetectable (Figure S18).

The photocurrent response to light pulses also mirrors the current‐time behavior described in Chidsey's seminal potential‐step studies: a brief anodic transient at light‐on (charging of the electrochemical double layer), followed by a cathodic transient at light‐off (discharge).^[^ ^35^ ^]^ When the complete reaction mixture is present, the photocurrent adopts an ideal square‐wave profile, indicating that the current arises exclusively from non‐thermal electron transfer.^[^ ^39^, ^40^ ^]^ To corroborate this, the temperature at both the electrode and in the solution was monitored using a thermocouple. Figure S19 shows that the solution temperature remained nearly constant throughout the reaction, in contrast to the electrode temperature, which responded directly to the laser pulses. Using Newton's law of cooling (Equation S1), we estimated that the electrode temperature can reach approximately 40 °C (Figure S20). When the reaction was performed in the dark at 40 °C, no photocurrent was detected, indicating that heat alone is not sufficient to catalyze the reaction. Moreover, the electrode's temperature response was identical with and without Au NPs, suggesting that the observed heating arises from the laser itself rather than from any plasmonic thermal contribution. Finally, the differences in photocatalytic performance between bases cannot be attributed to electrode composition, homogeneity, or stability, since all reactions were performed with the same electrode. Photostability was further verified by re‐testing the electrode at the end of the series using the initial reaction mixture with 2,6‐dimethoxypyridine as the base. As shown in Figures S21 and S22, the photocurrent responses at the beginning and end of the experiments nearly overlap, indicating only minimal electrode degradation under both illumination conditions, likely limited to minor Au NP leaching. Importantly, the extracted reaction rates remain unchanged, underscoring the robustness of the system and the reliability of the mechanistic insights derived from these measurements.

(1)

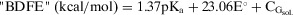




Analysis of the rate constants (Figures S23–S36 and Tables S1–S14) and steady‐state photocurrents reveals that catalytic activity is maximized when pyridine is used as the base. As noted by the Knowles group,^[^ ^5^ ^]^ optimal reactivity is achieved when the “BDFE” matches the BDFE of the bond being activated. The “BDFE” is calculated using Equation 1,^[^ ^41^ ^]^ which combines the acidity of the base (p*K*_a_), the oxidant strength (*E* ^○^), and a solvent‐dependent parameter ($C_{G_{sol.}}$), which for acetonitrile is 54.9 kcal·mol^−1^.^[^ ^8^ ^]^ As aforementioned, the C─H bond BDFE of BNAH in acetonitrile is 86.9 kcal·mol^−1^,^[^ ^3^ ^]^ which, according to Knowles’ proposal, allows estimation of the average hot‐hole energy involved in the catalytic process. This is particularly significant because plasmonic excitation first produces a nonFermi–Dirac distribution of carriers that subsequently thermalizes into a high‐temperature Fermi–Dirac distribution with transient variations in energy and population.^[^ ^13^, ^16^, ^17^ ^]^ As a result, the energy of the hot carriers driving catalysis is a parameter that is typically inaccessible.

Using the pyridine p*K*_a_ from Table 1, the average plasmonic oxidation potential was estimated as ∼0.64 V versus Fc⁺/Fc (∼1.04 V versus SCE), corresponding to holes located at 5.72 eV on the absolute (vacuum) scale. The work function of gold is well established, typically ranging from 5.1–5.3 eV depending on crystal orientation, cleanliness, and measurement method. Taking a representative mid‐value of 5.2 eV relative to vacuum,^[^ ^42^ ^]^ the average hot‐hole energy driving catalysis is therefore ∼0.52 eV, which lies well within the range expected for gold.^[^ ^43^ ^]^ Using this value, the electron‐driving force can be estimated from Equation 2,^[^ ^1^ ^]^ where *F* is Faraday's constant and 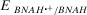 = 0.57 V versus SCE.^[^ ^26^, ^31^ ^]^ The resulting − Δ*G* ^○^_*ET*_ was calculated to be 0.47 eV.

(2)

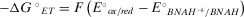




Mechanistic elucidation of PCET reactions is inherently challenging, as the common diagnostic tools each have important limitations. Intermediates are often difficult to detect, either because they do not accumulate to spectroscopically observable levels or because their signals overlap with those of other species, making their absence an unreliable argument for a concerted mechanism. Similarly, kinetic isotope effects can sometimes provide misleading information, since values close to unity may be observed even when proton transfer is involved in the rate‐determining step. A more robust approach relies on analyzing the driving‐force dependence of the reaction rate constant.^[^ ^1^ ^]^

(3) $$\;-\Delta G_{PT}^{\circ }=1.37\left(pK_{a\left(H^{+}base\right)}-pK_{a\left(BNAH^{\cdot +}\right)}\right)$$

Distinct mechanistic pathways, stepwise ET–PT, stepwise PT–ET, or concerted CEPT, exhibit characteristic dependencies of their rate constants on the underlying electron‐ and proton‐transfer driving forces. In a stepwise mechanism, the observed rate is governed by the driving force of the initial ET or PT event. If this first step is rate‐limiting, the rate increases with the moderate, Marcus‐type dependence expected for classical charge‐transfer reactions. If the first step instead establishes a rapid pre‐equilibrium, the overall rate becomes proportional to the corresponding equilibrium constant, resulting in a much steeper dependence on driving force. In contrast, in a concerted electron–proton transfer the reaction draws simultaneously on the driving forces of both ET and PT, lowering the effective barrier and producing a weaker, more symmetric sensitivity to variations in oxidant or base strength. Consequently, examining how the rate varies with − Δ*G* ^○^_*ET*_ and $-\Delta G_{PT}^{\circ }$ provides a powerful diagnostic to distinguish ET‐limited, PT‐limited, pre‐equilibrium, and genuinely concerted CEPT pathways, as discussed elsewhere.^[^ ^1^ ^]^

(4)

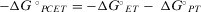




The use of bare plasmonic particles makes it difficult to tune the driving force for electron transfer; therefore, the analysis was restricted to proton exchange. The value of $-\Delta G_{PT}^{\circ }$ was estimated from Equation 3, and the − Δ*G* ^○^_*PCET*_ from Equation 4.^[^ ^1^ ^]^ The results are summarized in Table 1. The driving‐force dependence of the reaction rate constant is presented in Figure 2 for both illumination powers. In both cases, the rate constant increases linearly with $-\Delta G_{PT}^{\circ }$, reaching a maximum with pyridine. Beyond this point, further increases in the proton‐driving force result in a decrease in the rate constant, consistent with the predictions of Knowles.^[^ ^5^ ^]^ Linear fits of the data up to pyridine gave slopes of 2.7 ± 0.6 eV^−1^ (*R* ^2^ = 0.92) and 3.2 ± 0.9 eV^−1^(*R* ^2^ = 0.86), using 50% and 60% laser attenuation, respectively. Such moderate slopes are characteristic of an ET–PT mechanism, which requires pre‐association of the base and is therefore sensitive to its p*K*_a_, and consequently to the driving force.^[^ ^1^ ^]^ The differences in slope lie within the estimated uncertainties and therefore cannot be interpreted as a change in the underlying mechanism. Further reduction of the laser attenuation introduced accuracy issues in the photocurrent detection.

**Figure 2:**
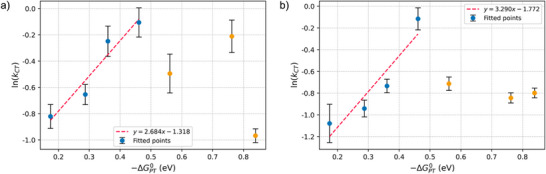
Dependence of the reaction rate constant on the proton‐transfer driving force in BNAH oxidation: a) 50% laser attenuation; b) 60% laser attenuation.

In conclusion, this work demonstrates that plasmonic energy‐filter electrodes offer a powerful and broadly applicable platform for driving and dissecting proton‐coupled electron transfer processes. Through controlled extraction of high‐energy hot carriers, plasmon excitation in gold nanoparticles provides sufficient driving force to activate bonds with strengths exceeding 85 kcal·mol^−1^. In the model system studied here, BNAH undergoes C─H bond activation through an ET–PT mechanism that requires pre‐association of the base, leading to formation of the neutral radical and subsequent oxidation (Figure 3). The hot holes mediating this transformation possess an average energy of ∼0.52 eV, showcasing the capacity of plasmonic systems to access highly energetic redox steps. Beyond this specific example, the approach establishes a general strategy for probing fundamental PCET behavior, mapping driving‐force dependencies, and distinguishing between competing mechanistic regimes. More broadly, the ability to photogenerate carriers with tunable and well‐defined energies positions plasmonic materials as versatile light‐harvesting elements for the development of new photoredox transformations and for mechanistic interrogation of challenging bond‐activation pathways.

**Figure 3:**
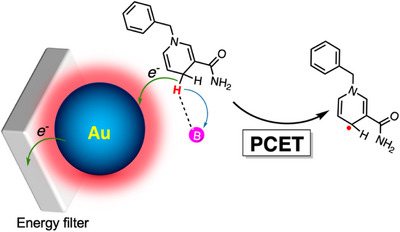
Schematic representation of C─H bond activation in BNAH via oxidative MS–PCET, mediated by plasmonic nanoparticles.

## Conflict of Interests

The authors declare no conflict of interest.

## Supporting information



Supporting Information

## Data Availability

The data that support the findings of this study are available from the corresponding author upon reasonable request.

## References

[1] R. Tyburski , T. Liu , S. D. Glover , L. Hammarström , J. Am. Chem. Soc. 2021, 143, 560–576, 10.1021/jacs.0c09106. 33405896 PMC7880575

[2] J. M. Mayer , Acc. Chem. Res. 2011, 44, 36–46. 20977224 10.1021/ar100093z PMC3022952

[3] J. J. Warren , T. A. Tronic , J. M. Mayer , Chem. Rev. 2010, 110, 6961–7001. 20925411 10.1021/cr100085k PMC3006073

[4] R. R. Knowles , E. N. Jacobsen , Proc. Natl. Acad. Sci. USA 2010, 107, 20678–20685, 10.1073/pnas.1006402107. 20956302 PMC2996434

[5] E. C. Gentry , R. R. Knowles , Acc. Chem. Res. 2016, 49, 1546–1556, 10.1021/acs.accounts.6b00272. 27472068 PMC5102158

[6] C. M. Morton , Q. Zhu , H. Ripberger , L. Troian‐Gautier , Z. S. D. Toa , R. R. Knowles , E. J. Alexanian , J. Am. Chem. Soc. 2019, 141, 13253–13260. 31356059 10.1021/jacs.9b06834 PMC6856561

[7] T. F. Markle , J. W. Darcy , J. M. Mayer , Sci. Adv. 2018, 4, eaat5776. 30027119 10.1126/sciadv.aat5776 PMC6044737

[8] G. J. Choi , R. R. Knowles , J. Am. Chem. Soc. 2015, 137, 9226–9229, 10.1021/jacs.5b05377. 26166022 PMC4643263

[9] H. G. Yayla , H. Wang , K. T. Tarantino , H. S. Orbe , R. R. Knowles , J. Am. Chem. Soc. 2016, 138, 10794–10797, 10.1021/jacs.6b06517. 27515494 PMC5110324

[10] J. D. Megiatto , A. Antoniuk‐Pablant , D. Gust , T. A. Moore , A. L. Moore , Proc. Natl. Acad. Sci. USA 2012, 109, 15578–15583, 10.1073/pnas.1118348109. 22566659 PMC3465380

[11] R. S. Khnayzer , P. A. Liddell , A. L. Moore , T. A. Moore , D. Gust , J. Am. Chem. Soc. 2013, 135, 14068–14070, 10.1021/ja407816f. 24028290

[12] J. Heyrovsky , J. Electroanal. Chem. 1963, 6, 82–93.

[13] M. L. Brongersma , N. J. Halas , P. Nordlander , Nat. Nanotechnol. 2015, 10, 25–34, 10.1038/nnano.2014.311. 25559968

[14] C. Clavero , Nat. Photonics 2014, 8, 95–103, 10.1038/nphoton.2013.238.

[15] S. Linic , P. Christopher , D. B. Ingram , Nat. Mater. 2011, 10, 911–921, 10.1038/nmat3151. 22109608

[16] A. Wach , R. Bericat‐Vadell , C. Bacellar , C. Cirelli , P. J. M. Johnson , R. G. Castillo , V. R. Silveira , P. Broqvist , J. Kullgren , A. Maximenko , T. Sobol , E. Partyka‐Jankowska , P. Nordlander , N. J. Halas , J. Szlachetko , J. Sá , Nat. Commun. 2025, 16, 2274, 10.1038/s41467-025-57657-1. 40050628 PMC11885627

[17] P. Nordlander , J. Aizpurua , ACS Photonics 2019, 6, 3020–3031.

[18] P. Christopher , S. Linic , Nat. Chem. 2011, 3, 467–472. 21602862 10.1038/nchem.1032

[19] R. Bericat‐Vadell , P. Sekar , Y. Patehebieke , X. Zou , N. Kaul , P. Broqvist , R. Lindblad , A. Lindblad , A. Arkhypchuk , C.‐J. Walletin , J. Sá , Mater. Today Chem. 2023, 34, 101783.

[20] S. Fukuzumi , O. Inada , T. Suenobu , J. Am. Chem. Soc. 2003, 125, 4808–4816, 10.1021/ja029623y. 12696900

[21] T. Matsuo , J. M. Mayer , Inorg. Chem. 2005, 44, 2150–2158, 10.1021/ic048170q. 15792449

[22] P. Sekar , R. Bericat‐Vadell , Y. Patehebieke , P. Bröqvist , C.‐J. Wallentin , M. Görlin , J. Sá , Nano Lett. 2024, 24, 8619–8625, 10.1021/acs.nanolett.4c01803. 38973705 PMC11261604

[23] R. Bericat‐Vadell , J. Sá , Small Struct. 2025, 6, 2500185, 10.1002/sstr.202500185.

[24] J. Fast , U. Aeberhard , S. P. Bremmer , H. Linke , Appl. Phys. Rev. 2021, 8, 021309, 10.1063/5.0038263.

[25] M. F. Lichterman , S. Hu , M. H. Richter , E. J. Crumlin , S. Axnanda , M. Favaro , W. Drisdell , Z. Hussain , T. Mayer , B. S. Brunschwig , N. S. Lewis , Z. Liu , H.‐J. Lewerenz , Energy Environ. Sci. 2015, 8, 2409–2416, 10.1039/C5EE01014D.

[26] R. Charaf , PhD thesis, Photoinduced and Proton‐Coupled Electron Transfer Mechanisms of Photoredox Catalysis. Uppsala University, 2025, https://www.uu.se/en/events/defences/2025‐05‐09‐rima‐charaf‐photoinduced‐and‐proton‐coupled‐electron‐transfer‐mechanisms‐of‐photoredox‐catalysis (Accessed on 15/08/2025).

[27] F. G. Bordwell , J. A. Harrelson , T.‐Y. Lynch , J. Org. Chem. 1990, 55, 3337–3341, 10.1021/jo00297a064.

[28] P. Krtil , L. Kavan , P. Novák , J. Electrochem. Soc. 1993, 140, 3390–3395, 10.1149/1.2221100.

[29] X. An , D. Stelter , T. Keyes , B. M. Reinhard , Chem 2019, 5, 2228–2242.

[30] S. Liu , Z. Wu , Z. Zhu , K. Feng , Y. Zhou , X. Hu , X. Huang , B. Zhang , X. Dong , Y. Ma , K. Nie , J. Shen , Z. Wang , J. He , J. Wang , Y.u Ji , B. Yan , Q. Zhang , A. Genest , X. Zhang , C. Li , B.o Wu , X. An , G. Rupprechter , L. e He , Nat. Commun. 2025, 16, 2245, 10.1038/s41467-025-57569-0. 40050268 PMC11885817

[31] F. Strieth‐Kalthoff , F. Glorius , Chem 2020, 6, 1888–1903.

[32] Y. Kim , J. G. Smith , P. K. Jain , Nat. Chem. 2018, 10, 763–769, 10.1038/s41557-018-0054-3. 29736005

[33] J. Ouyang , A. J. Bard , J. Phys. Chem. 1987, 91, 4058–4062, 10.1021/j100299a025.

[34] S. Tshepelevitsh , A. Kütt , M. Lõkov , I. Kaljurand , J. Saame , A. Heering , P. G. Plieger , R. Vianello , I. Leito , Eur. J. Org. Chem. 2019, 2019, 6735–6748, 10.1002/ejoc.201900956.

[35] C. E. D. Chidsey , Science 1991, 251, 919–922, 10.1126/science.251.4996.919. 17847385

[36] V. S. Conceição , D. P. M. Saraiva , G. Denuault , M. Bertotti , Anal. Chem. 2024, 96, 14766–14774. 39226461 10.1021/acs.analchem.4c01645 PMC11411494

[37] J. Yuasa , S. Yamada , S. Fukuzumi , J. Am. Chem. Soc. 2006, 128, 14938–14948, 10.1021/ja064708a. 17105305

[38] A. Anne , P. Haplot , J. Moiroux , P. Neda , J. M. Saveant , J. Am. Chem. Soc. 1992, 114, 4694–4701, 10.1021/ja00038a036.

[39] M. Maley , J. W. Hill , P. Saha , J. D. Walmsley , C. M. Hill , J. Phys. Chem. C 2019, 123, 12390–12399, 10.1021/acs.jpcc.9b01479.

[40] A. J. Bagnall , S. Ganguli , A. Sekretareva , Angew. Chem. Int. Ed. 2024, 63, e202314352, 10.1002/anie.202314352. 38009712

[41] R. G. Agarwal , S. C. Coste , B. D. Groff , A. M. Heuer , H. Noh , G. A. Parada , C. F. Wise , E. M. Nichols , J. J. Warren , J. M. Mayer , Chem. Rev. 2022, 122, 1–49, 10.1021/acs.chemrev.1c00521. 34928136 PMC9175307

[42] W. N. Hansen , G. J. Hansen , Surf. Sci. 2001, 481, 172–184, 10.1016/S0039-6028(01)01036-6.

[43] G. Tagliabue , J. S. DuChene , M. Abdellah , A. Habib , D. J. Gosztola , Y. Hattori , W.‐H. Cheng , K. Zheng , S. E. Canton , R. Sundararaman , J. Sá , H. A. Atwater , Nat. Mater. 2020, 19, 1312–1318. 32719510 10.1038/s41563-020-0737-1

