# Supplementary material for: Plasmonic Hot‐Carrier Redox Enables Proton‐Coupled Electron Transfer at C─H Bonds
Source: Angew Chem Int Ed Engl. 2025 Dec 17;65(6):e18818. doi: 10.1002/anie.202518818 (PMC12865242; doi:10.1002/anie.202518818)
Supplement: Supplementary file 1 — Supporting Information [file ANIE-65-e18818-s001.docx]

Supporting Information

Plasmonic Hot-Carrier Redox Enables Proton–Coupled Electron Transfer at C–H Bonds

Daniel Velev Latchev,^[a]^ Arthur Andreis,^[a]^ Julian Michael Heeg,^[a]^ and Jacinto Sá*^[a,b]^

[a] D. V. Latchev, J. M. Heeg, A. Andreis, Prof. J. Sá
Department of Chemistry-Ångström
Uppsala University
Uppsala, 751 20, Sweden
E-mail: jacinto.sa@kemi.uu.se

[b] Prof. J. Sá
Institute of Physical Chemistry, Polish Academy of Sciences
Warsaw, 01-224, Poland

EXPERIMENTAL & METHODS

Preparation of samples

*Fabrication of the energy-filter electrode with Au nanoparticles (NPs):*

Fluorine-doped tin oxide (FTO) conductive glass (NSG-Pilkington) pre-coated with a 10 nm amorphous TiO_2_ layer (conduction band edge ~4.4 eV) was used as the substrate for electrode preparation. Prior to deposition, the films were cleaned in a 2% Helmax aqueous solution under ultrasonic agitation for 30 minutes, thoroughly rinsed with deionized water, and dried under a nitrogen stream to remove surface contaminants. To eliminate any remaining organic residues, the substrates were further treated with ozone for 15 minutes.

The cleaned TiO_2_-coated FTO glasses were then placed in a physical vapor deposition chamber (LEICA Instruments), where a thin gold layer (~2 nm) was deposited on the surface. The Au-coated films were annealed in a muffle furnace at 450 °C for 30 minutes and subsequently cooled to room temperature before being used in further experiments. The reproducibility of Au nanoparticle formation was assessed by monitoring the localized surface plasmon resonance band using UV–Vis spectroscopy and confirmed by X-ray photoelectron spectroscopy.

Characterisation of the electrodes

*UV-Vis measurements:* The UV-Vis spectra were collected using a Cary 5000 UV-VIS-NIR spectrophotometer.

*Scanning electron micrsocpy (SEM) measurements:* SEM was performed using a Zeiss Merlin microscope (Myfab, Ångström Laboratory). The acceleration voltage was 5 or 10 kV, and the working distance was between 5-7 mm.

*X-ray photoelectron spectrsocpy (XPS) measurements:* The XPS measurements were recorded using Al Ka (1487 eV) radiation and a Quantera II spectrometer from Physical Electronics (Myfab, Ångström Laboratory). The data were fitted using the doublet separation provided by the NIST XPS database. The charging was corrected using the advantageous C 1s peak, set to 284.6 eV.

Catalytic testing

Chronoamperometry experiments were performed in a 1.5 mL photoelectrochemical spectroscopy cell (Redox.me) using a two-electrode configuration, with the FTO/TiO_2_/Au NPs as the working electrode and a Pt wire as the counter electrode. The design of the cell can be found in the manufacturer website: <https://redox.me/collections/spectro-electrochemical-cells/products/spectro-ec-1-5-ml-spectro-electrochemical-cell>. The illuminated electrode area was 0.95 cm^2^, and we used a single photoelectrode as one of the windows. The electrolyte consisted of 100 mM tetrabutylammonium hexafluorophosphate (TBAPF_6_) in dry acetonitrile.

Plasmonic excitation was provided by a 635 nm laser (maximum power density: 130 mW·cm^-2^). Illumination was by 50-60% of the laser power using an optical filter and applied in square pulses of 2 s duration (0.5 Hz). Chronoamperometric measurements were conducted for 60 s, corresponding to 16 on–off cycles.

The reaction medium contained 2 mM 1-benzyl-1,4-dihydronicotinamide (BNAH, oxidation substrate), 100 mM TBAPF_6_ (supporting electrolyte), and 80 mM of the selected base. This concentration of base was chosen based on literature reports, where increasing the substrate-to-base ratio beyond 1:40 did not enhance the reaction rate.

A stock solution of BNAH (6.856 mg, 2 mM) and TBAPF_6_ (620 mg, 100 mM) in freshly dried acetonitrile was prepared (total volume: 16 mL). Aliquots were then used to prepare individual test solutions by adding the appropriate base concentration immediately before experiments, ensuring identical BNAH concentration across all measurements. For reproducibility checks, experiments were repeated with different vials (2 mL or 4 mL) to rule out glass effects. All solutions were prepared the day before use and stored for 16–18 h prior to measurement.

The experimental procedure was as follows:

1. The cell was assembled, the laser aligned with the electrode, and the oscillator configured (4 s period). The potentiostat was connected to the electrodes.
2. The test solution was introduced into the cell using a dedicated Pasteur pipette (one per base solution).
3. Chronoamperometry was carried out under pulsed illumination as described above.
4. After each run, the solution was removed using a separate pipette labeled for waste.
5. The photoelectrode was rinsed several times with acetonitrile electrolyte before introducing the new test solution.
6. Steps 2–5 were repeated for each specific reaction mixture while using the same electrode.

Chronoamperometry measurements were performed using a PalmSens EmStat4S potentiostat/galvanostat.

ADDITIONAL DATA

**Figure S1.** Schematic represenation of energy filter concept. Image adapted from elsewere. ^[^^[[1]](#endnote-1)]^


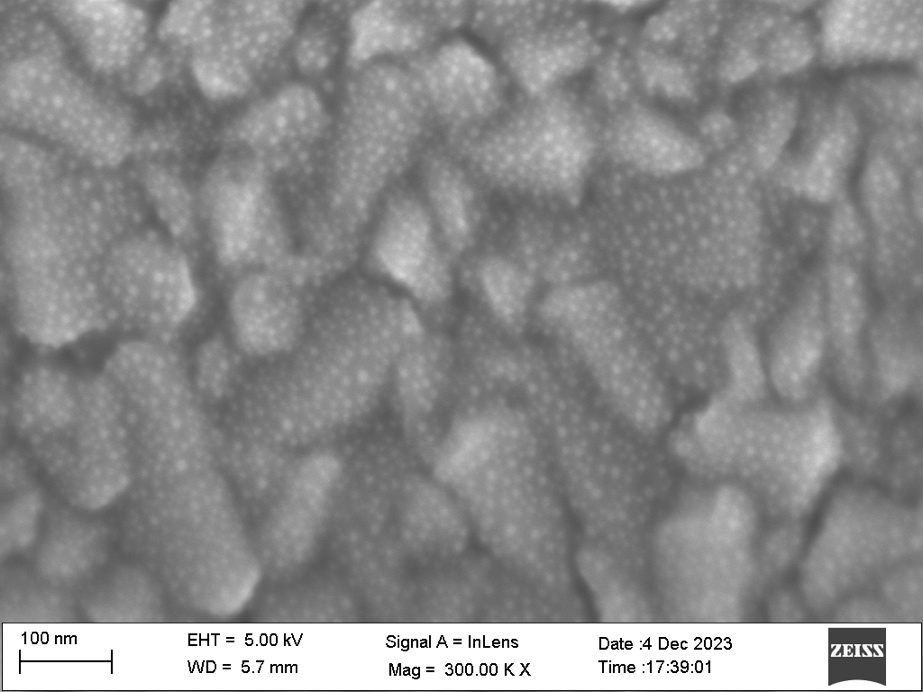


**a)**


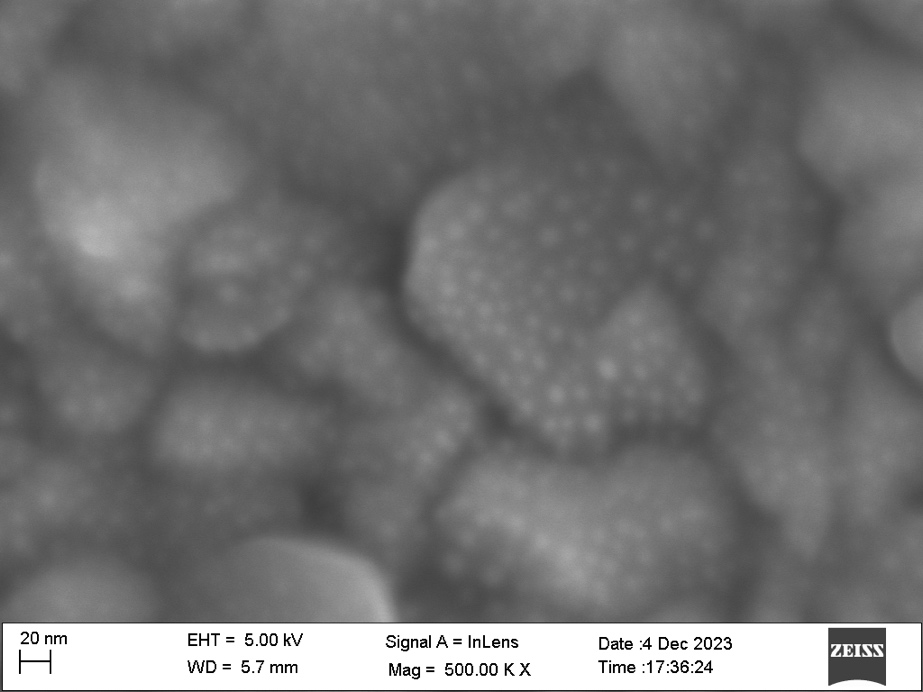


**b)**

**Figure S2.** SEM depicting the Au NPs on FTO\TiO_2_ after the annealing procedure: a) low magnification and b) high magnification.

Figure S3. Optical absorption of Au NPs on on FTO\TiO_2_.

**
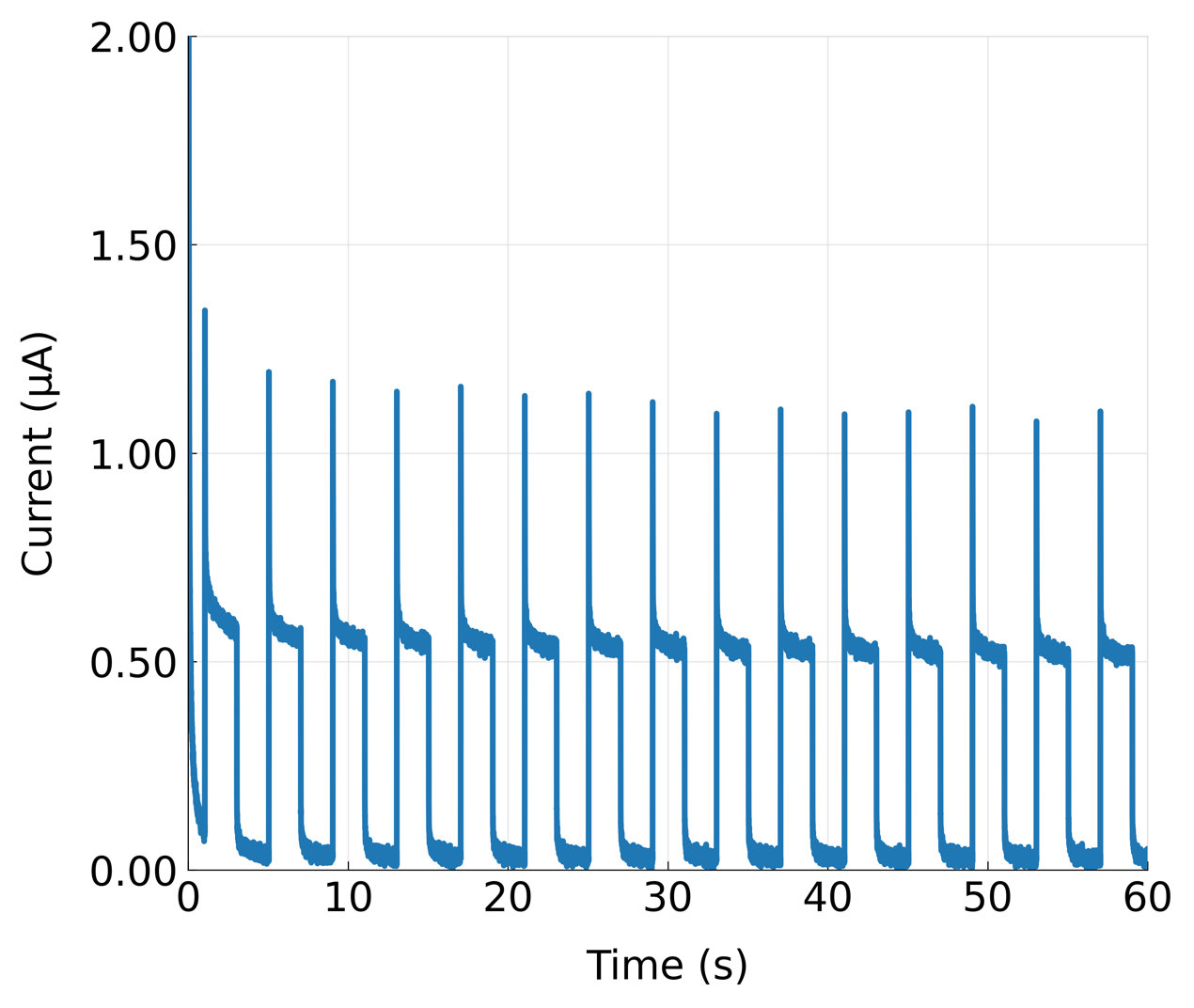
**

**Figure S4.** Chronoamperometry data of BNAH oxidation in the presence of 2,6-dimethoxypyridine with laser power attenuated by 50%.

**
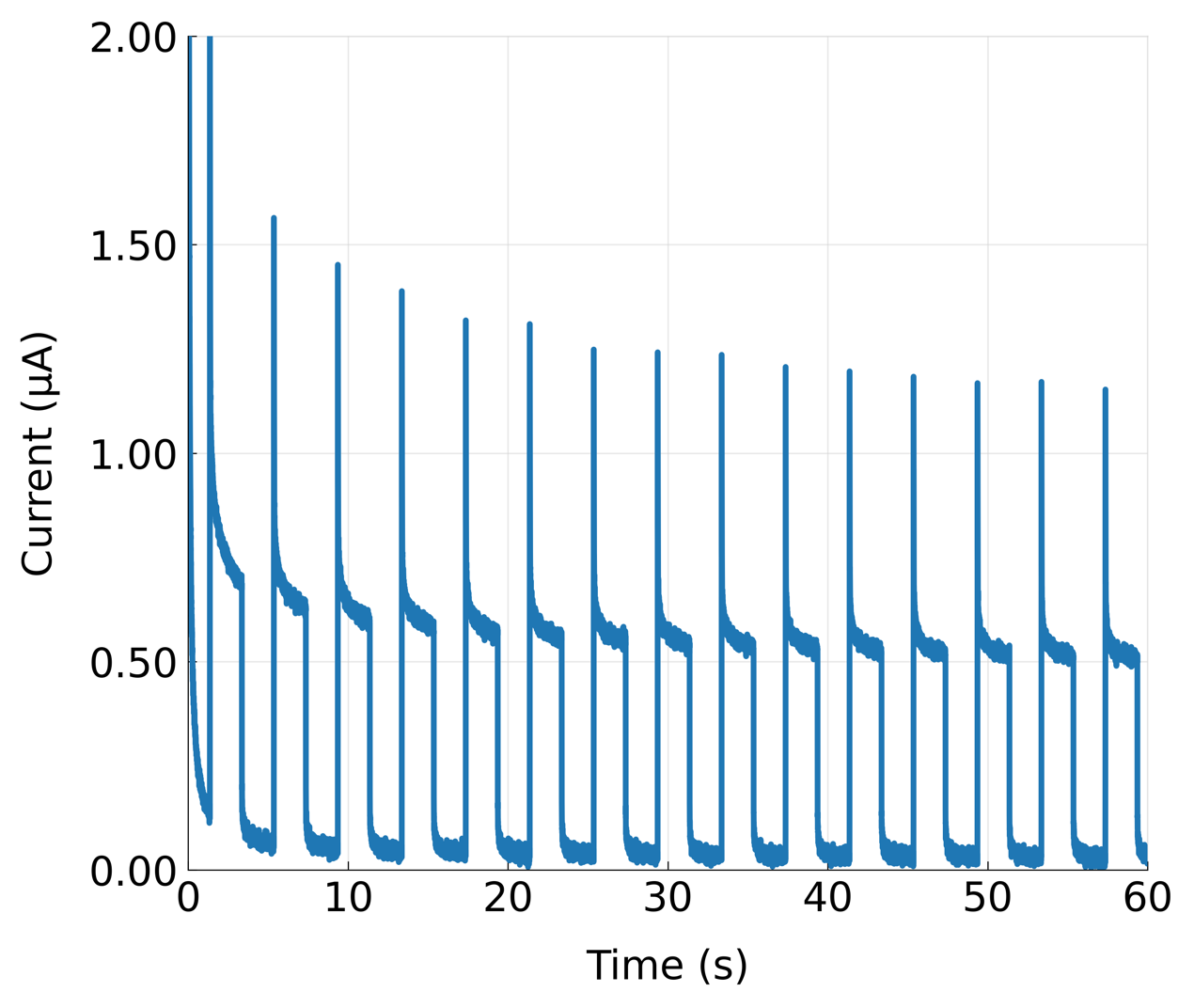
**

**Figure S5.** Chronoamperometry data of BNAH oxidation in the presence of 3-chloropyridine with laser power attenuated by 50%.

**
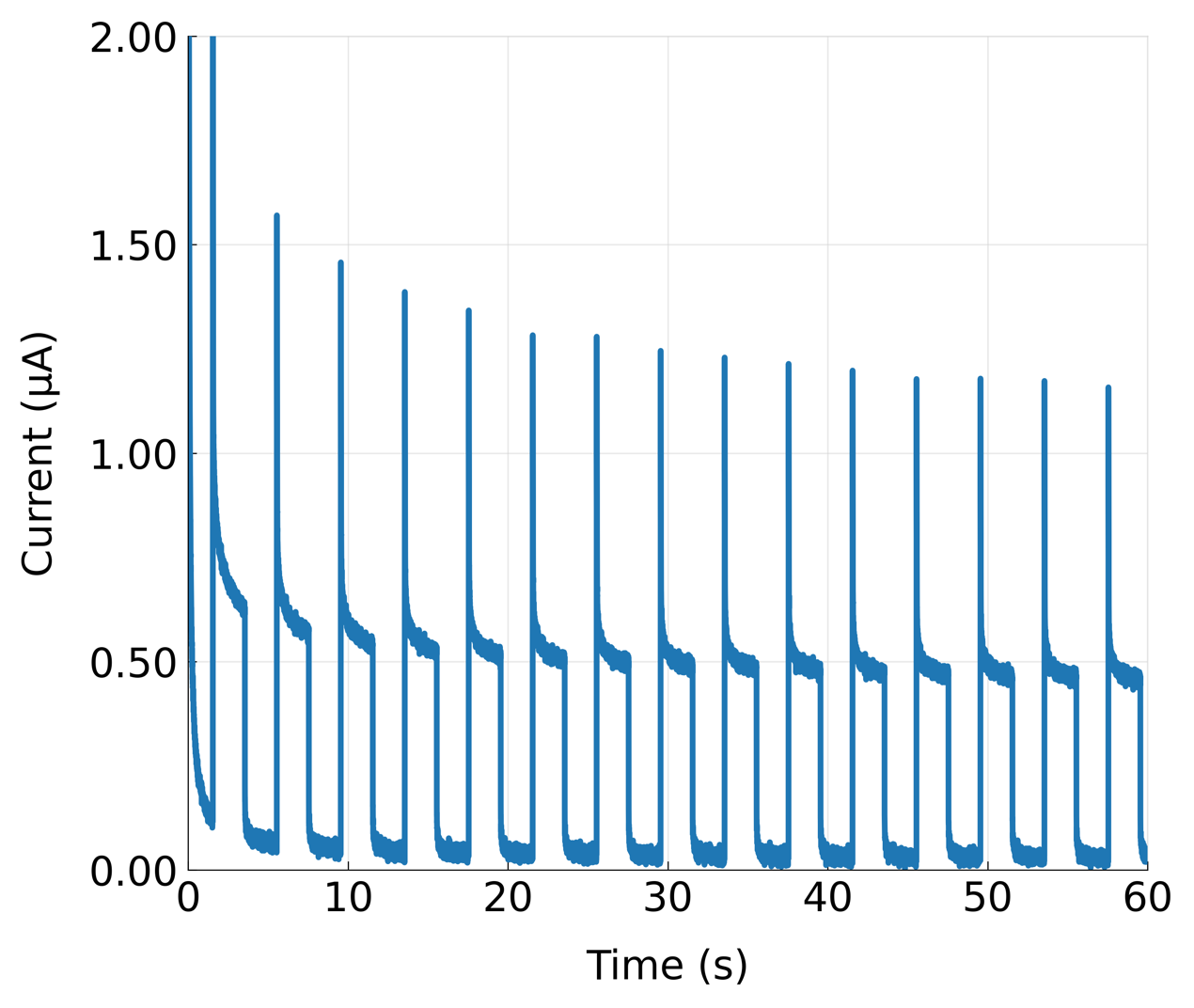
**

**Figure S6.** Chronoamperometry data of BNAH oxidation in the presence of 3-acetylpyridine with laser power attenuated by 50%.

**
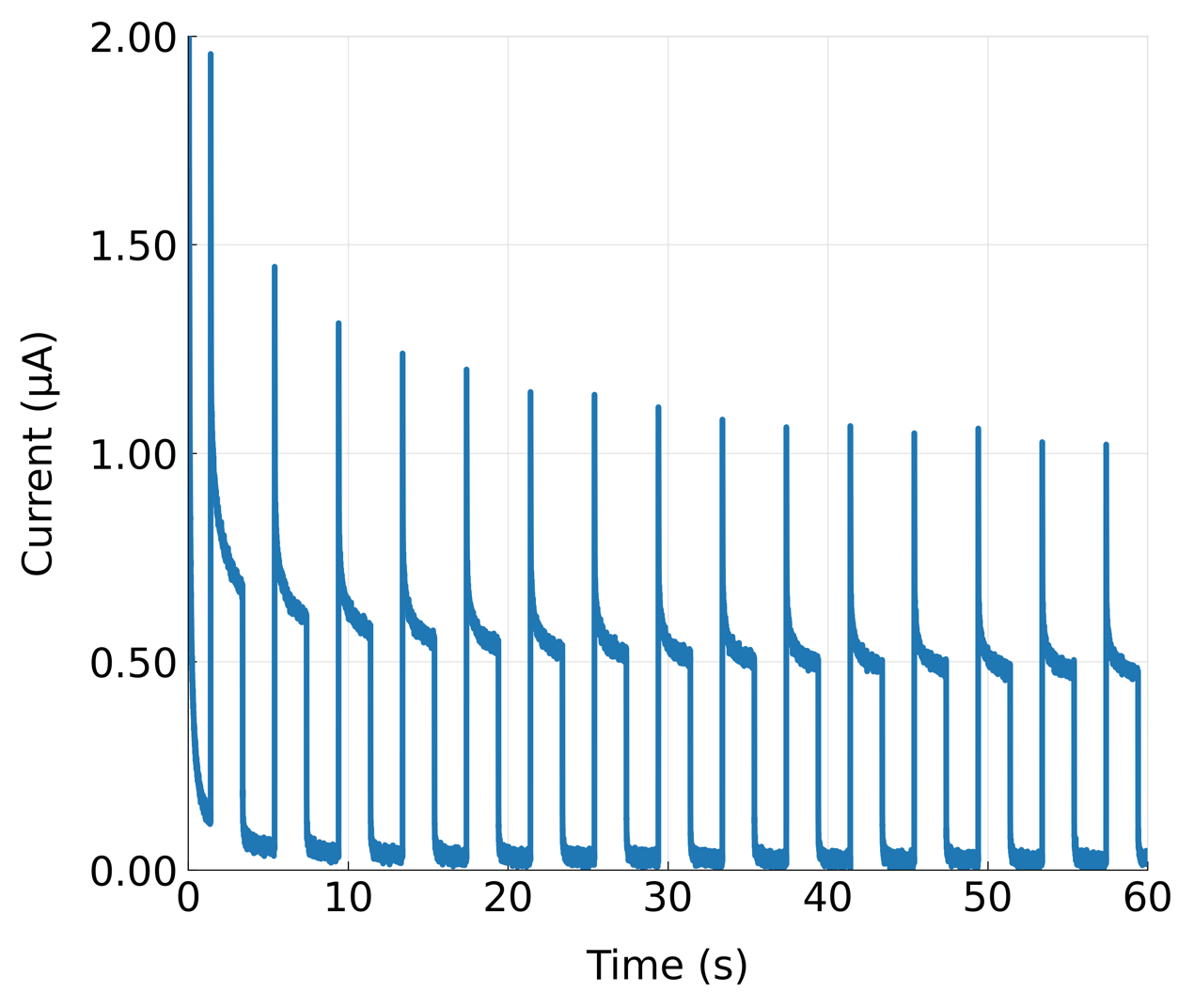
**

**Figure S7.** Chronoamperometry data of BNAH oxidation in the presence of pyridine with laser power attenuated by 50%.

**
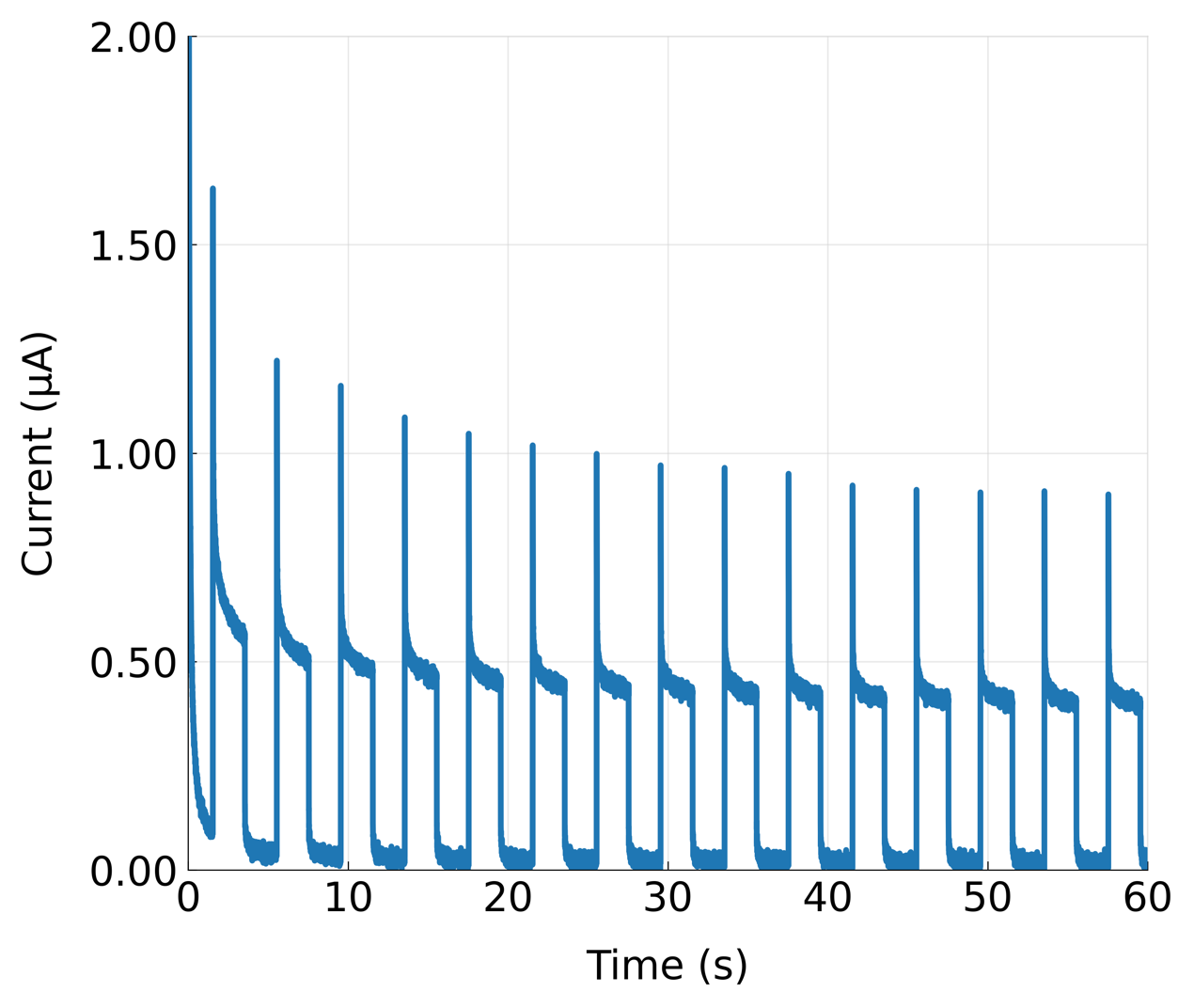
**

**Figure S8.** Chronoamperometry data of BNAH oxidation in the presence of 4-methoxypyridine with laser power attenuated by 50%.

**
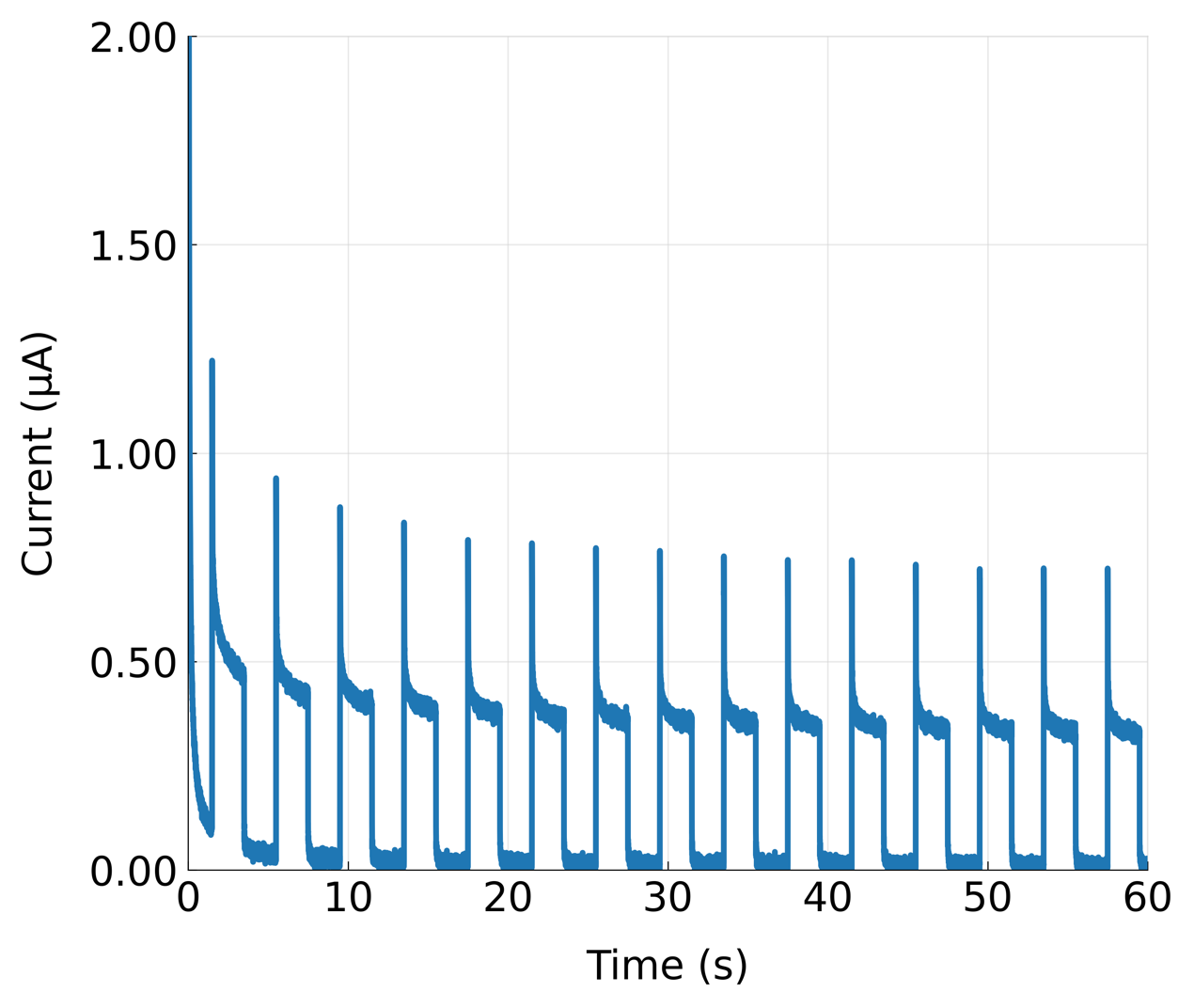
**

**Figure S9.** Chronoamperometry data of BNAH oxidation in the presence of 4-aminopyridine with laser power attenuated by 50%.

**
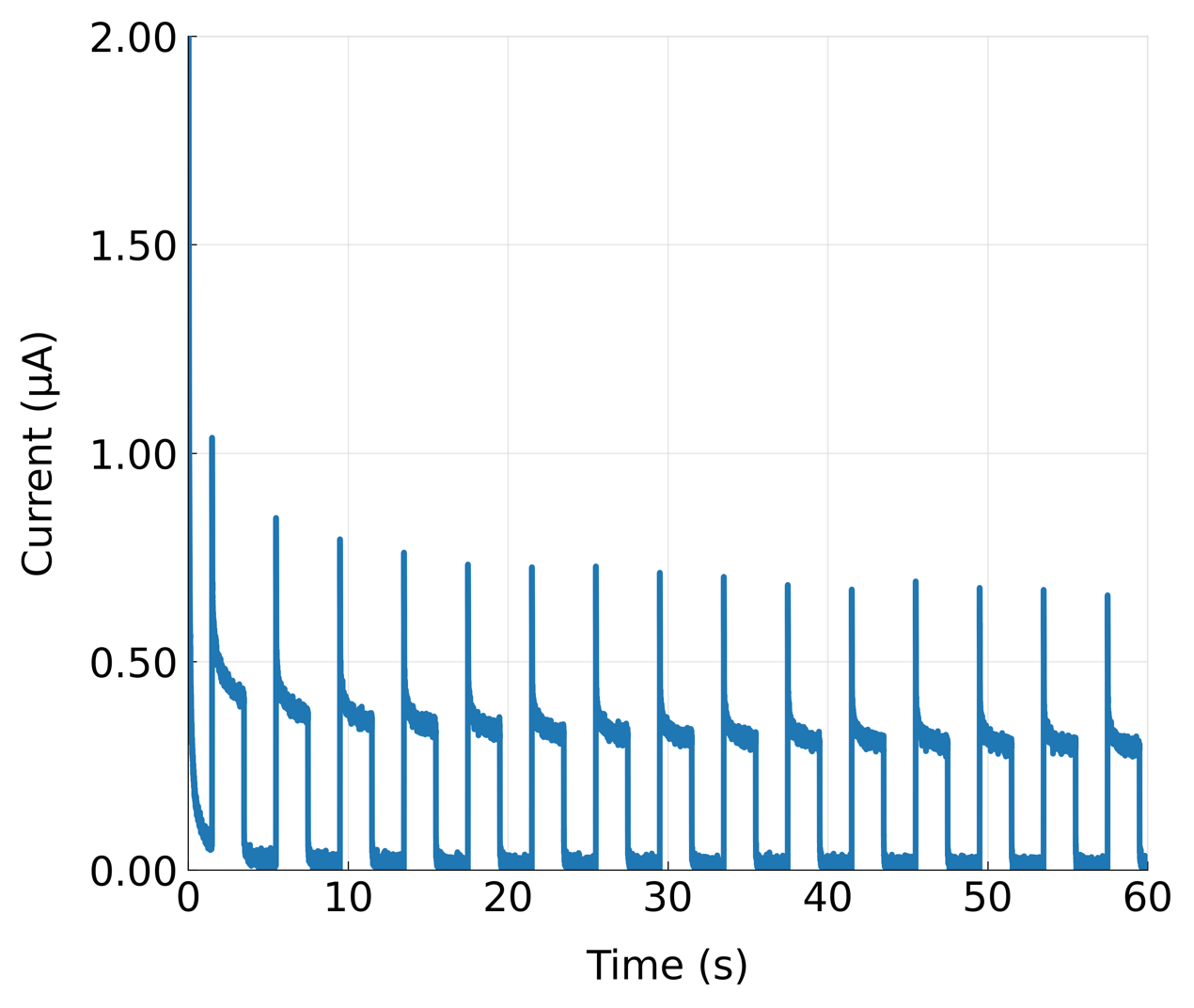
**

**Figure S10.** Chronoamperometry data of BNAH oxidation in the presence of piperidine with laser power attenuated by 50%.

**
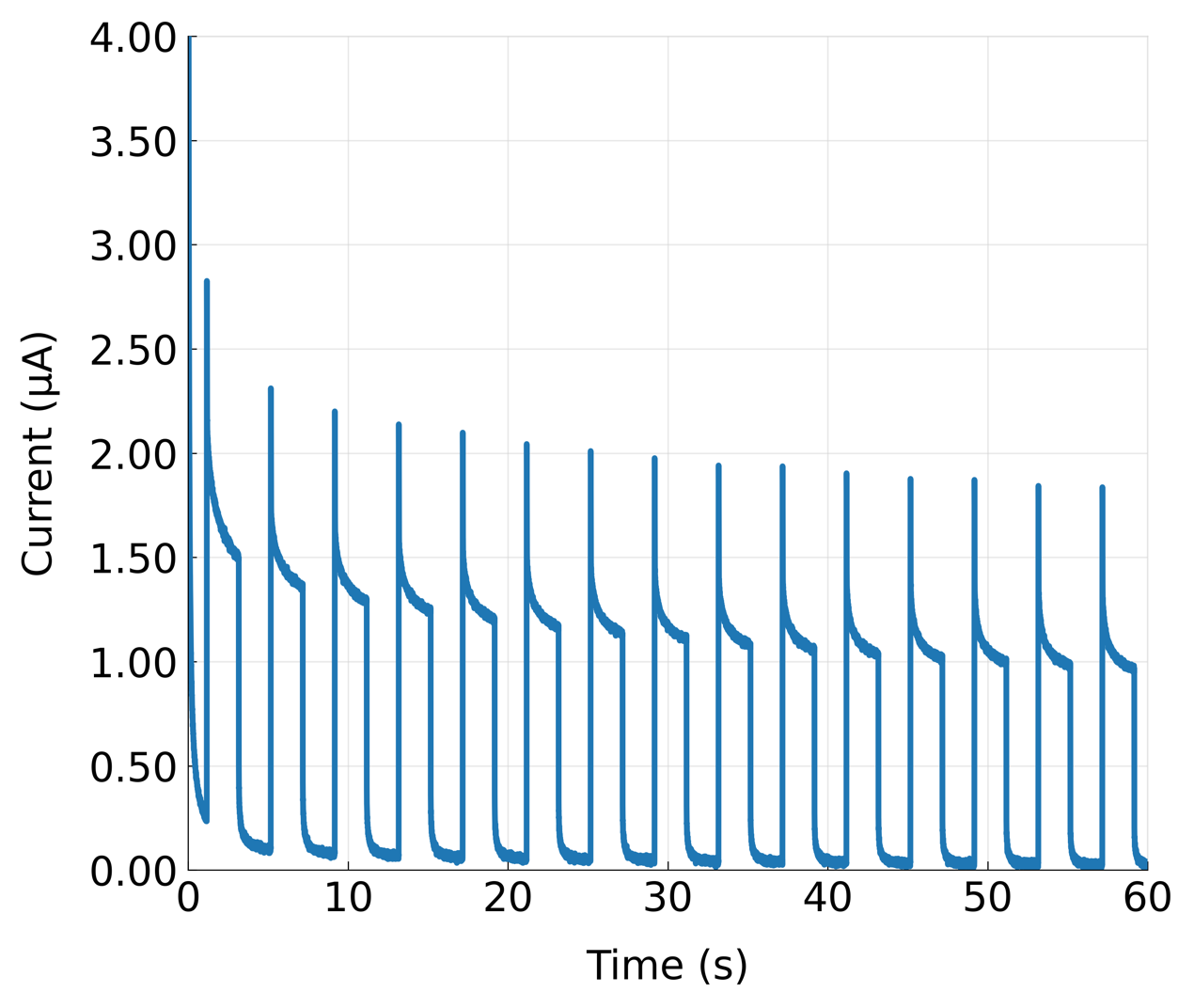
**

**Figure S11.** Chronoamperometry data of BNAH oxidation in the presence of 2,6-dimethoxypyridine with laser power attenuated by 60%.

**
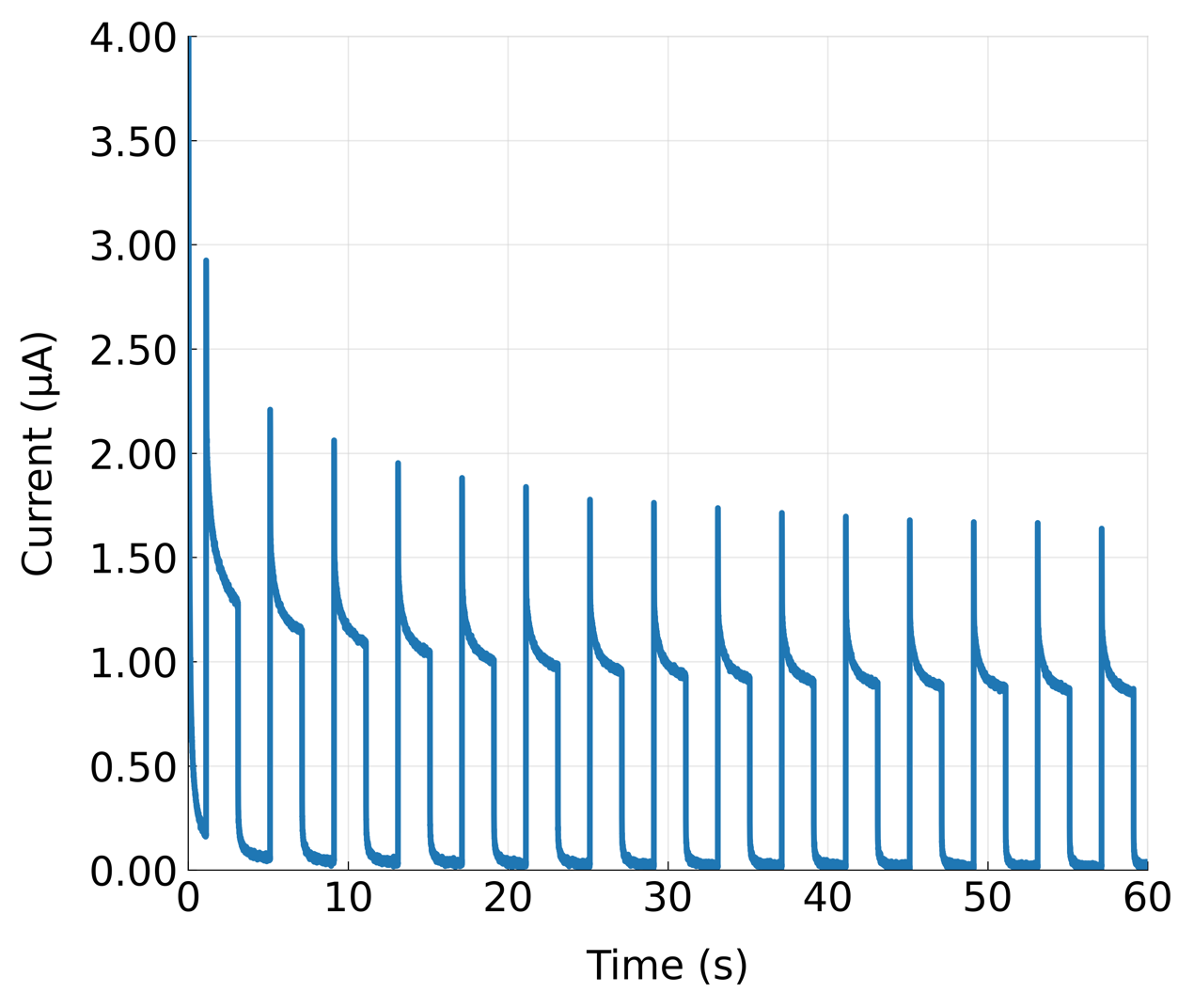
**

**Figure S12.** Chronoamperometry data of BNAH oxidation in the presence of 3-chloropyridine with laser power attenuated by 60%.

**
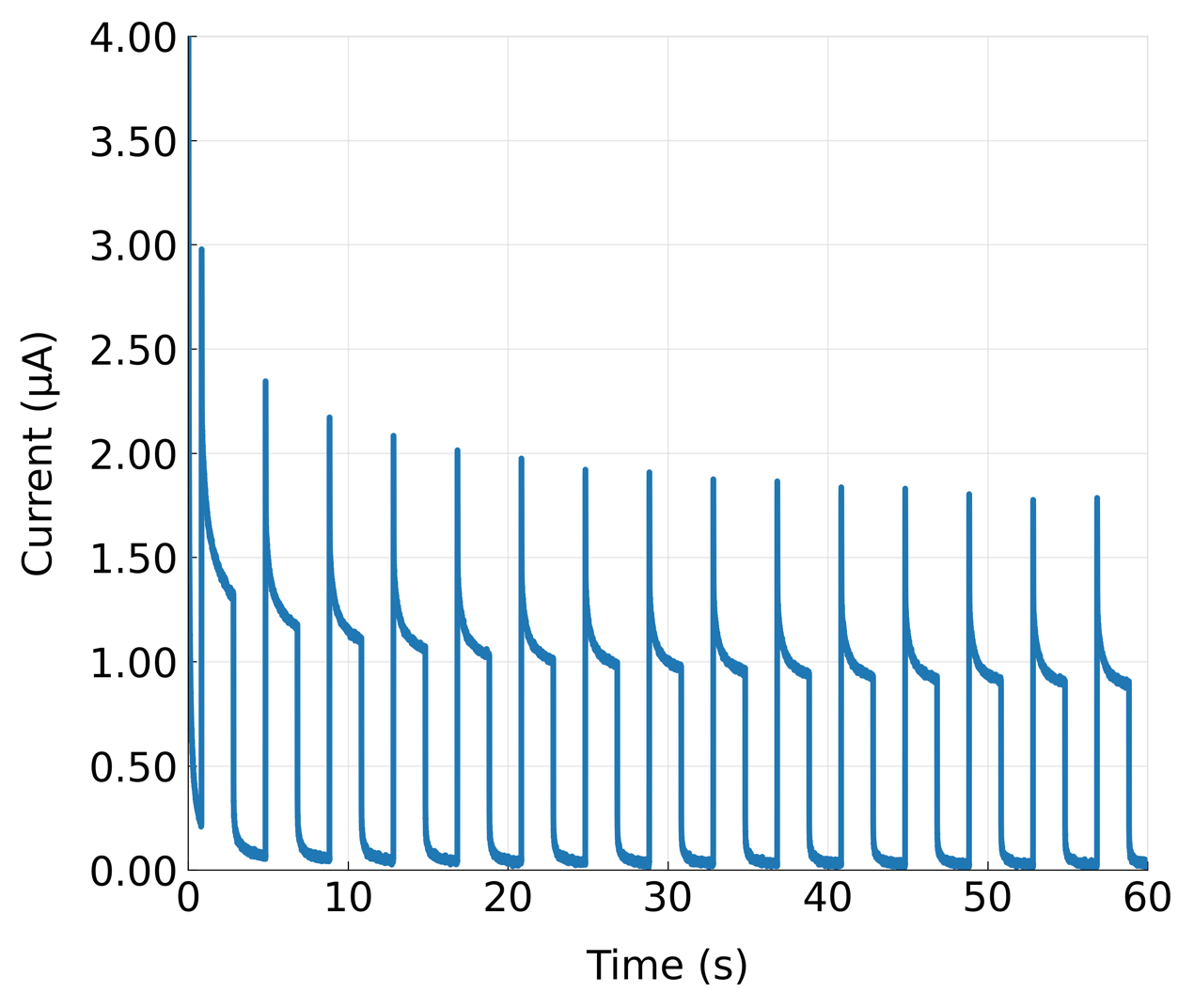
**

**Figure S13.** Chronoamperometry data of BNAH oxidation in the presence of 3-acetylpyridine with laser power attenuated by 60%.

**
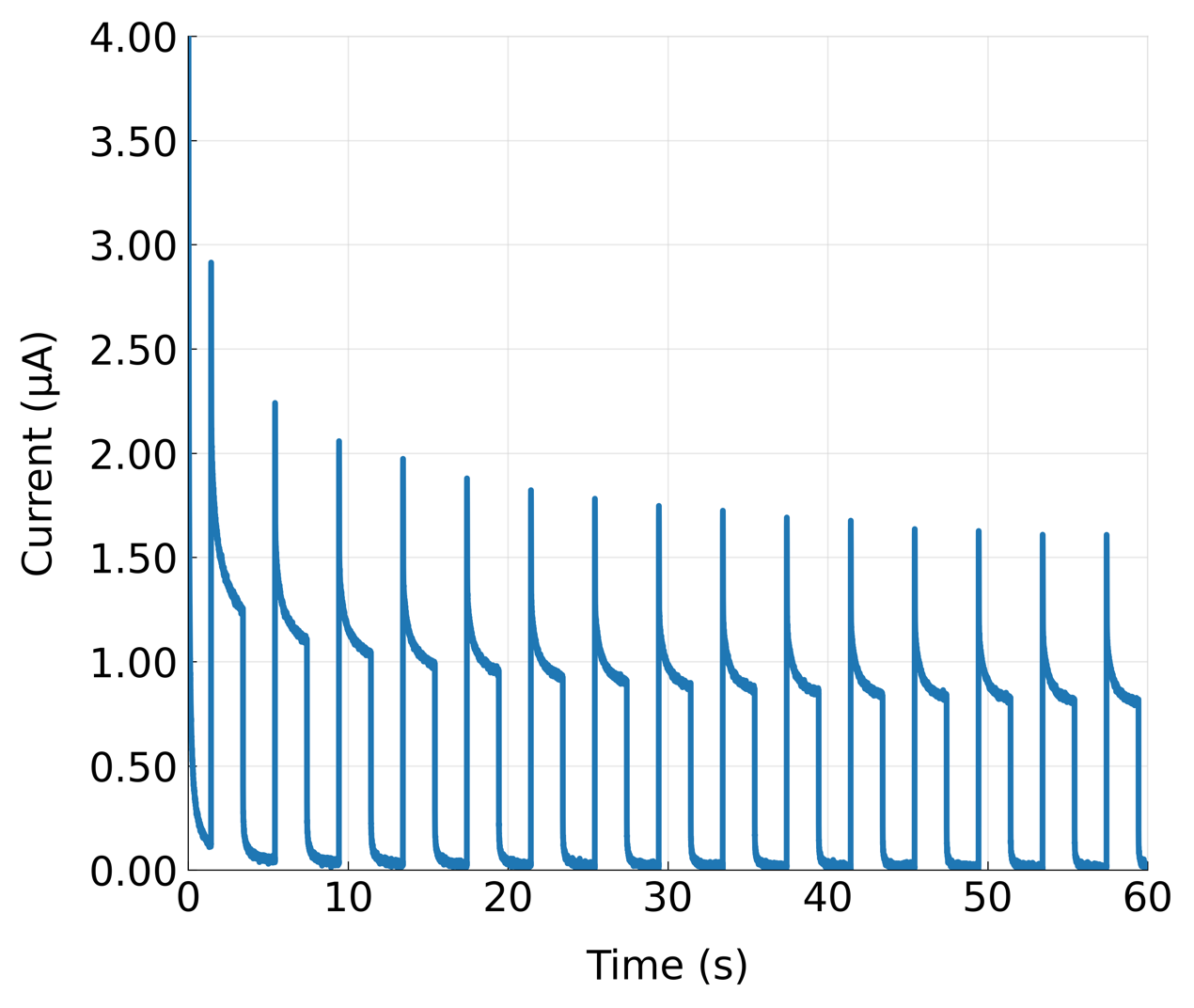
**

**Figure S14.** Chronoamperometry data of BNAH oxidation in the presence of pyridine with laser power attenuated by 60%.

**
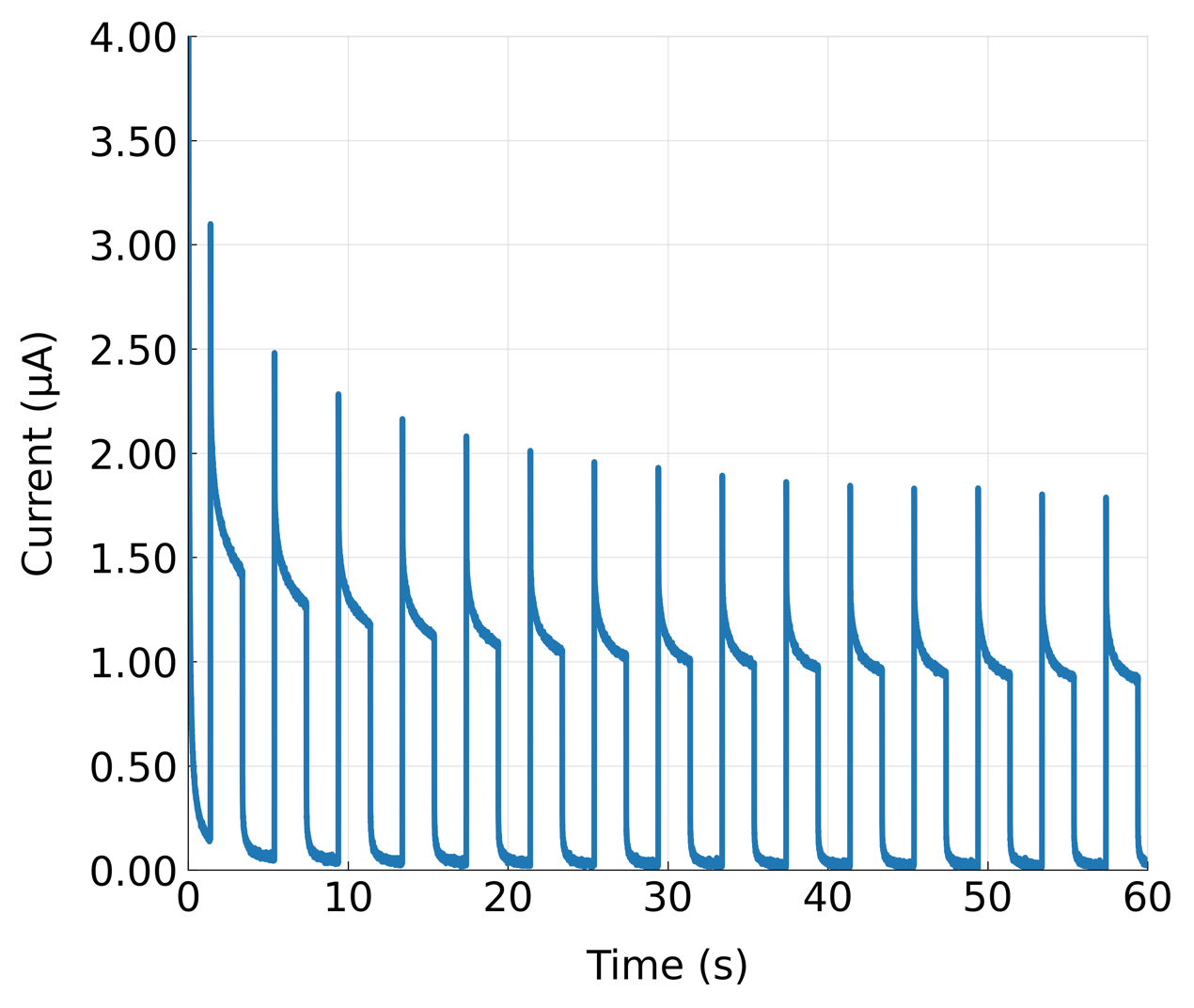
**

**Figure S15.** Chronoamperometry data of BNAH oxidation in the presence of 4-methoxypyridine with laser power attenuated by 60%.

**
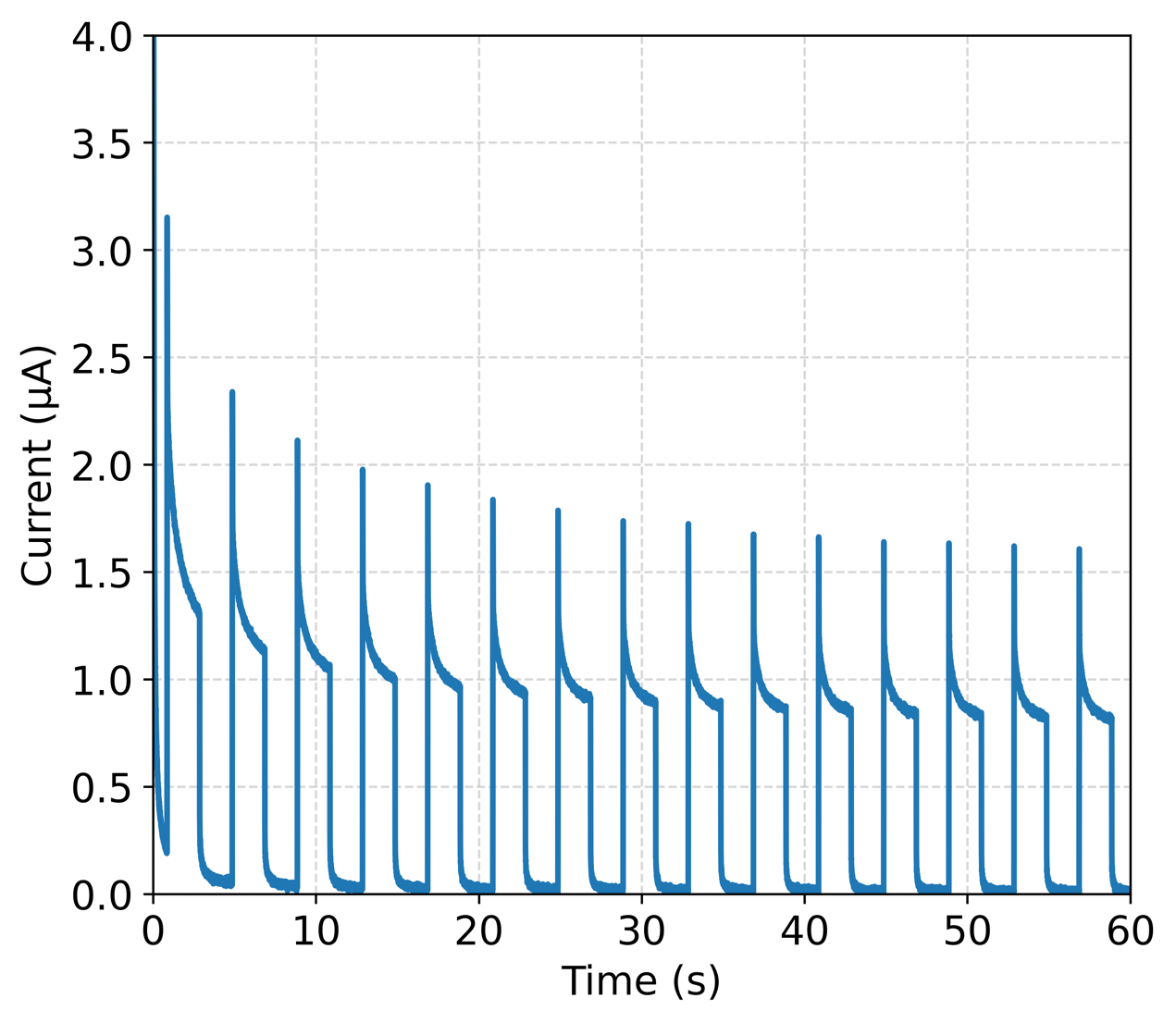
**

**Figure S16.** Chronoamperometry data of BNAH oxidation in the presence of 4-aminopyridine with laser power attenuated by 60%.

**
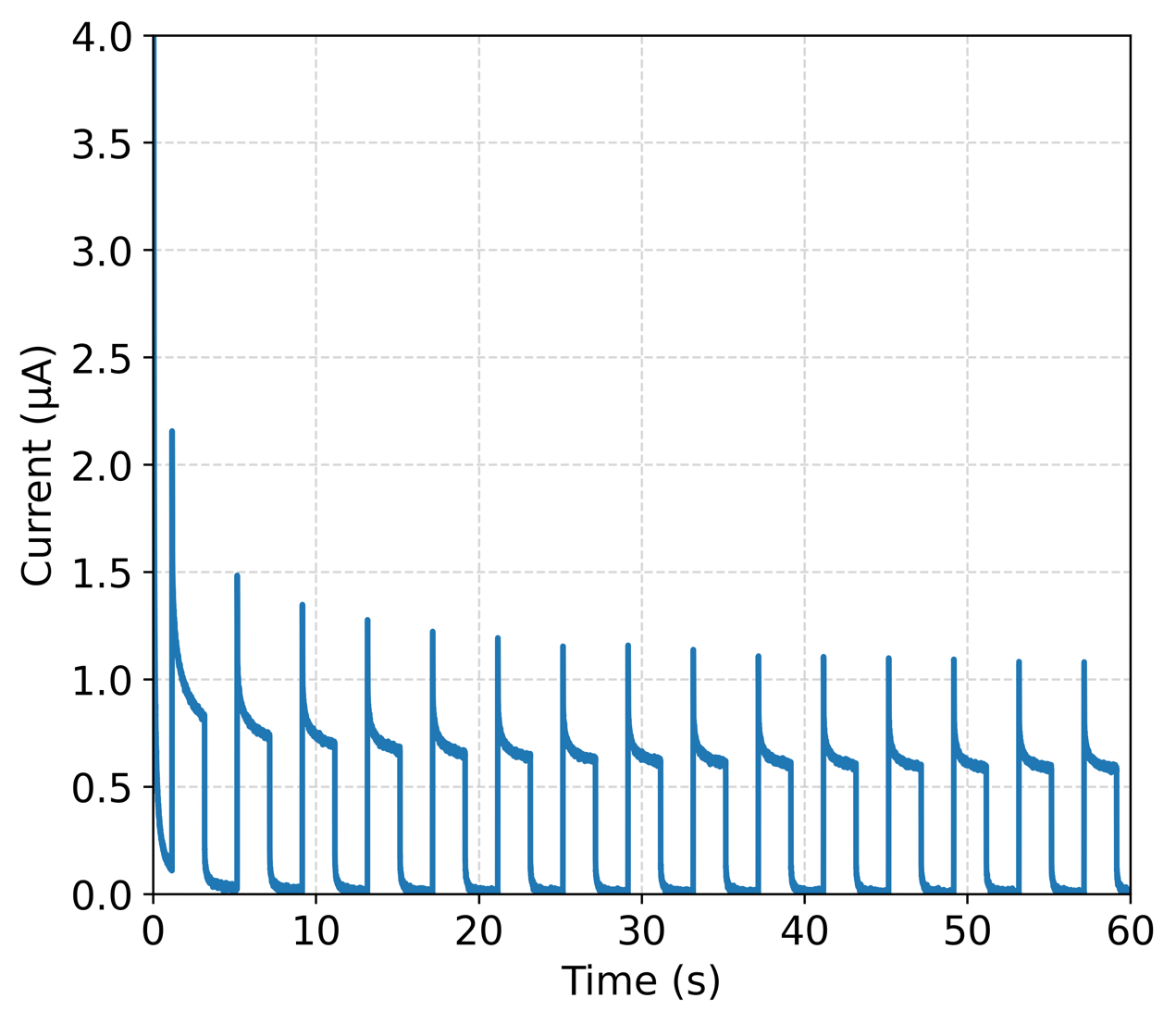
**

**Figure S17.** Chronoamperometry data of BNAH oxidation in the presence of piperidine with laser power attenuated by 60%.

**
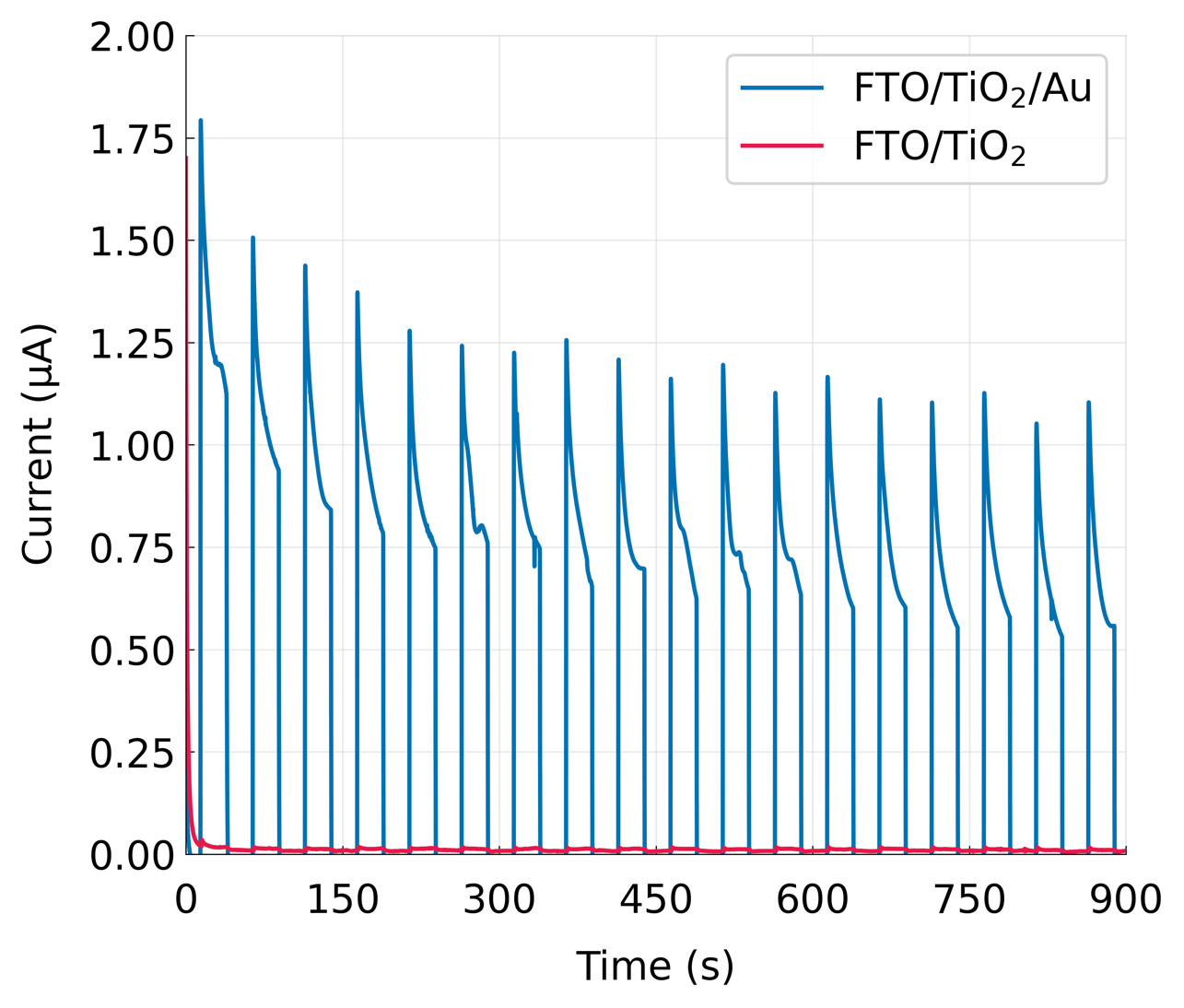
**

**Figure S18.** Chronoamperometry data of BNAH oxidation in the presence of 3-chloropyridine with laser power attenuated by 50%, comparing the effect of adding Au NPs to the electrode.

**
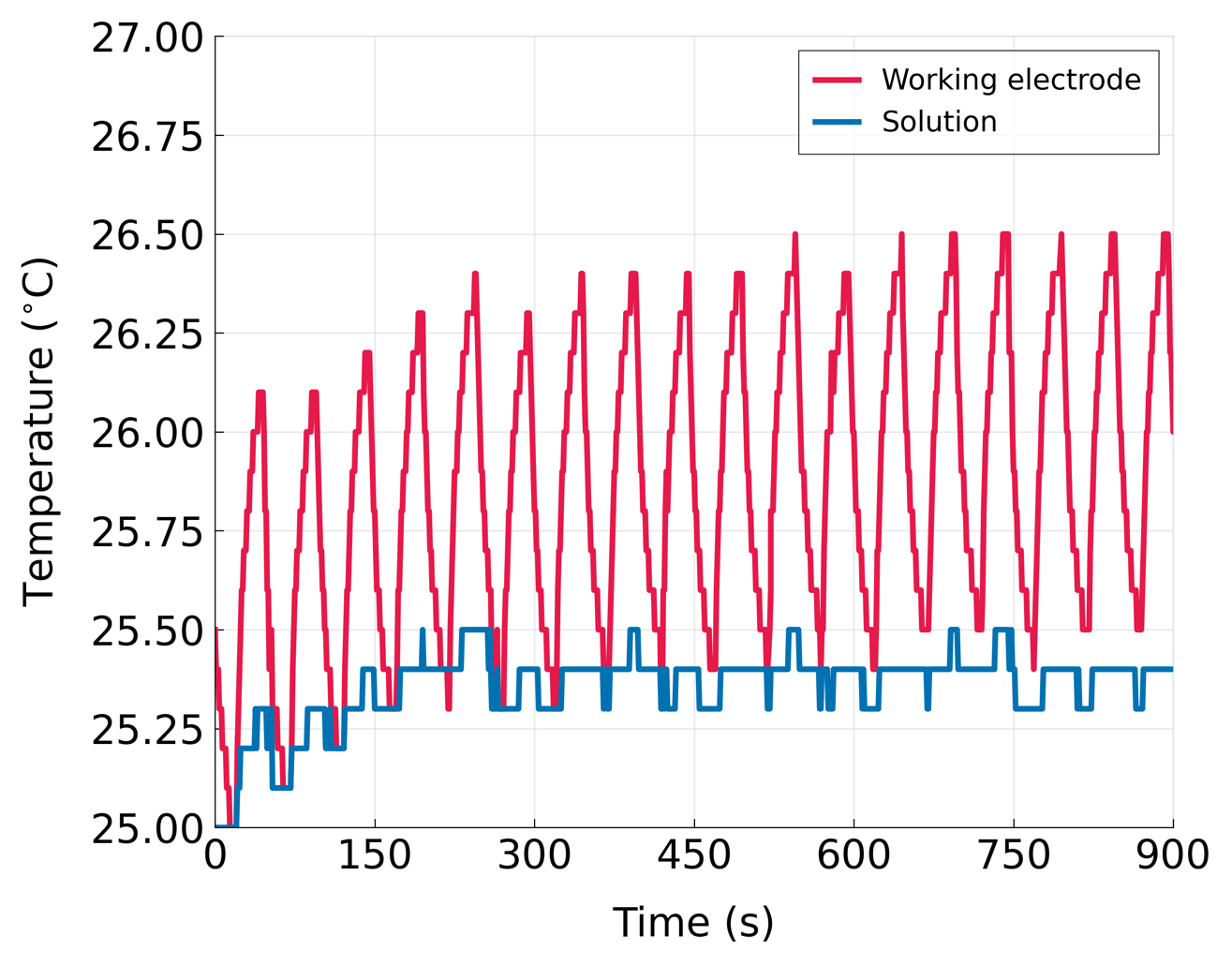
**

**Figure S19.** Temperature profiles during BNAH oxidation in the presence of 3-chloropyridine with laser power attenuated by 50%.

**
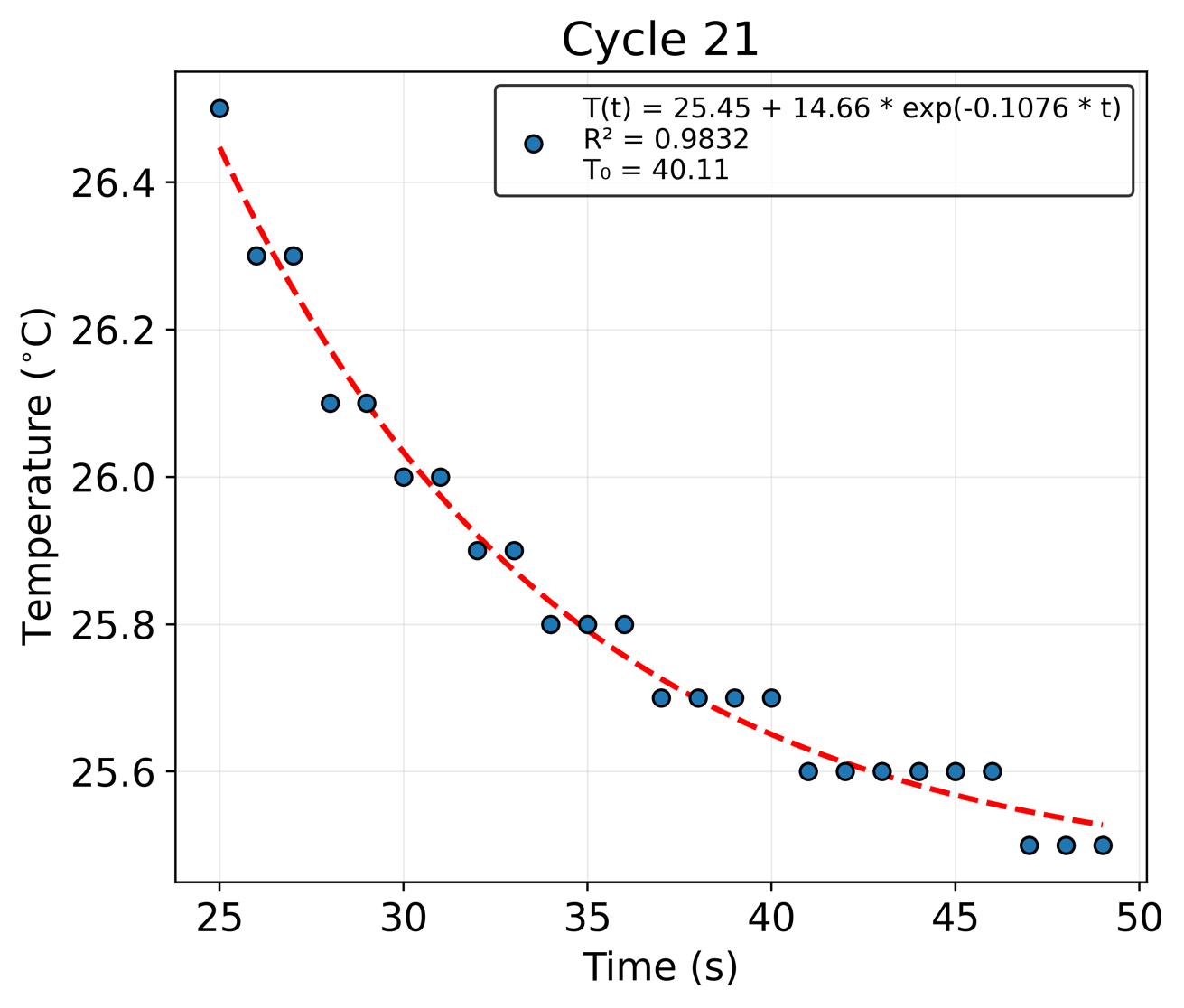
**

**Figure S20.** Fitting of one temperature cycle using Newton’s cooling law to extrapolate the maximum temperature reached at the electrode.

**Newton’s law of cooling**

$T\left( t \right)=T_{amb}+(T_{initial}-T_{amb})e^{-kt}$ eq. S1

$T\left( t \right)$ : The temperature of the object at a given time $t$

$T_{amb}$: The ambient or surrounding temperature

$T_{initial}$ : The initial temperature of the object at time $t=0$ (highest temperature of the electrode)

$k$ : A positive constant that depends on the object's properties (like its mass, specific heat, and surface area)

$t$ : Time

**
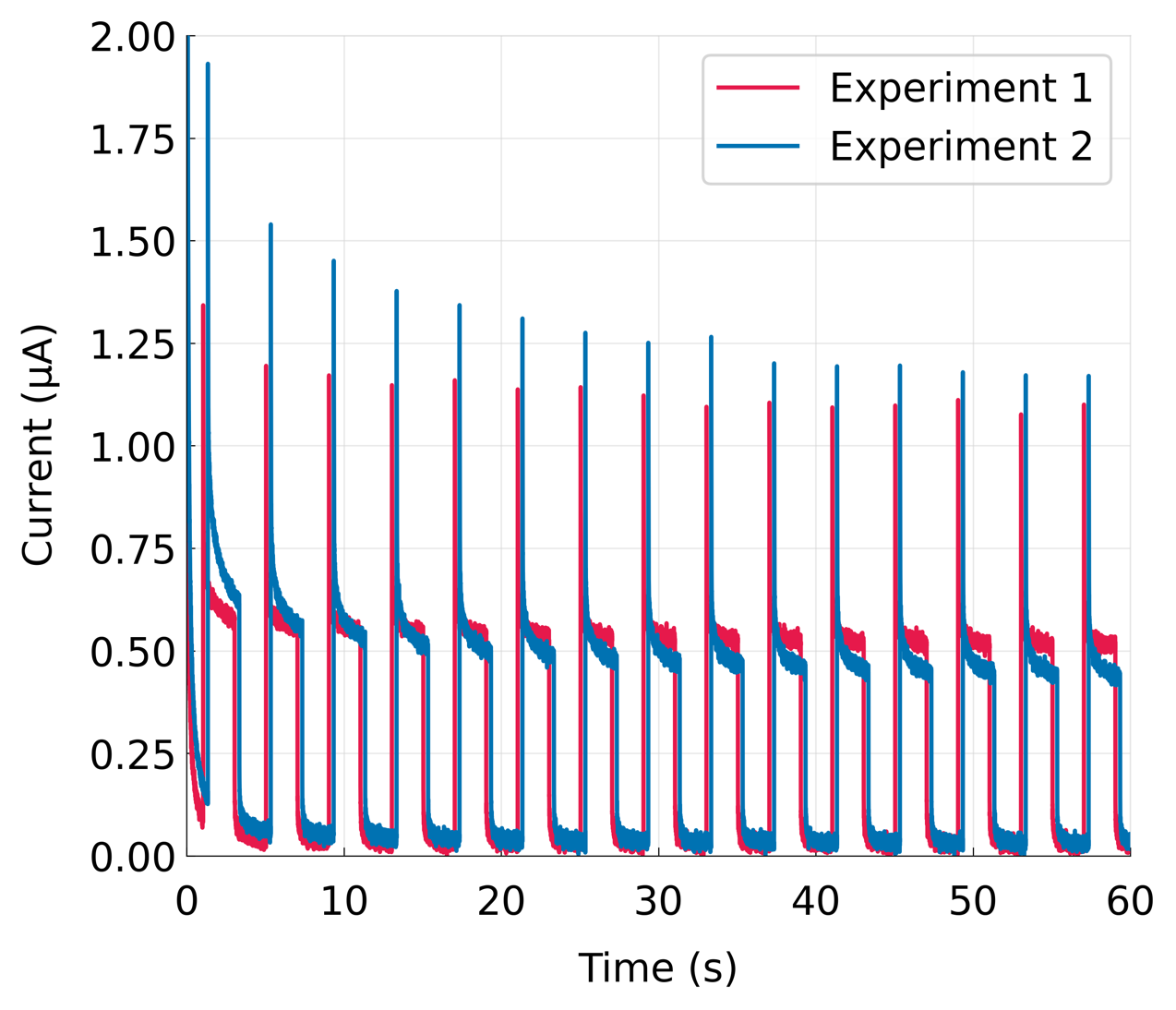
**

**Figure S21.** Chronoamperometry data of BNAH oxidation in the presence of 2,6-dimethoxypyridine with laser power reduced by 50%. The signals from two experiments using the same base, one at the beginning of the study and one after testing all bases, are compared to demonstrate the stability of the photoelectrode.

**
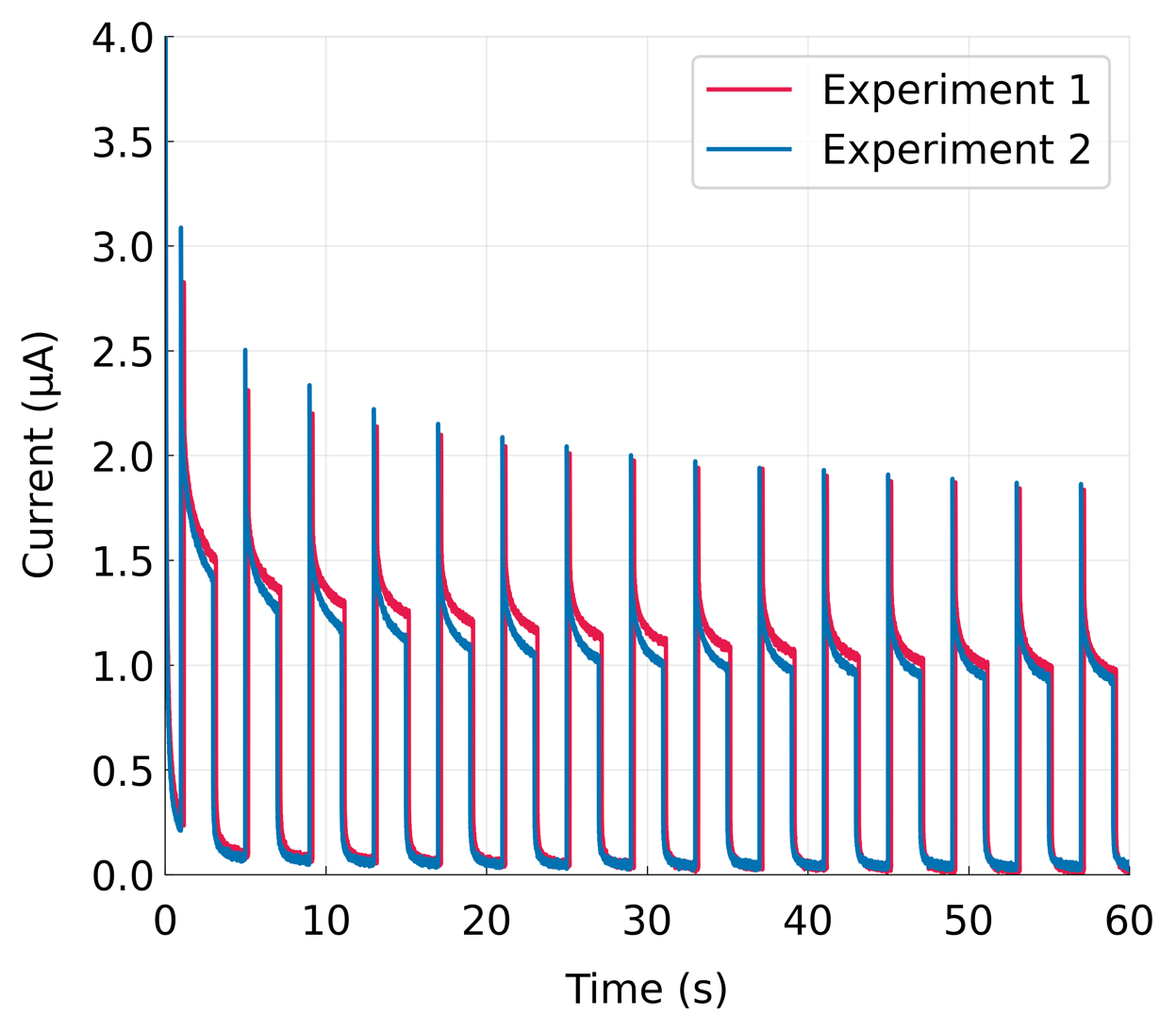
**

**Figure S22.** Chronoamperometry data of BNAH oxidation in the presence of 2,6-dimethoxypyridine with laser power reduced by 60%. The signals from two experiments using the same base, one at the beginning of the study and one after testing all bases, are compared to demonstrate the stability of the photoelectrode.

**
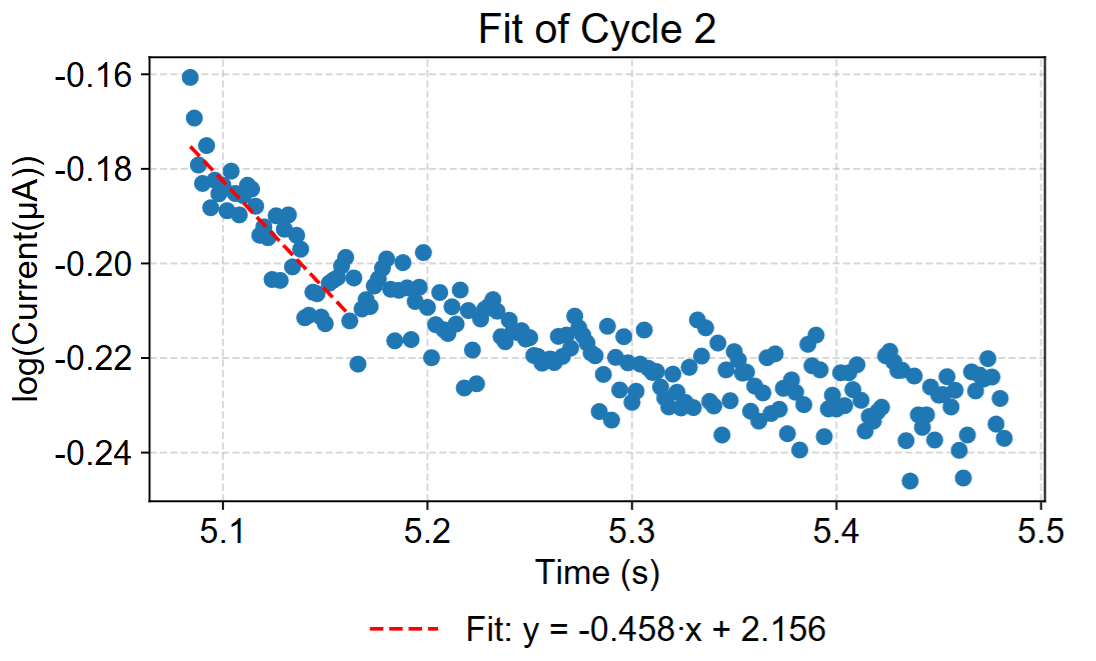
**

**Figure S23.** Fitting of the non-diffusion-limited region of the chronoamperometry data for BNAH oxidation in the presence of 2,6-dimethoxypyridine, with the laser power attenuated by 50%.

**Table S1.** Fitting parameters of the non-diffusion-limited region of the chronoamperometry data for BNAH oxidation in the presence of 2,6-dimethoxypyridine, with the laser power attenuated by 50%.

**
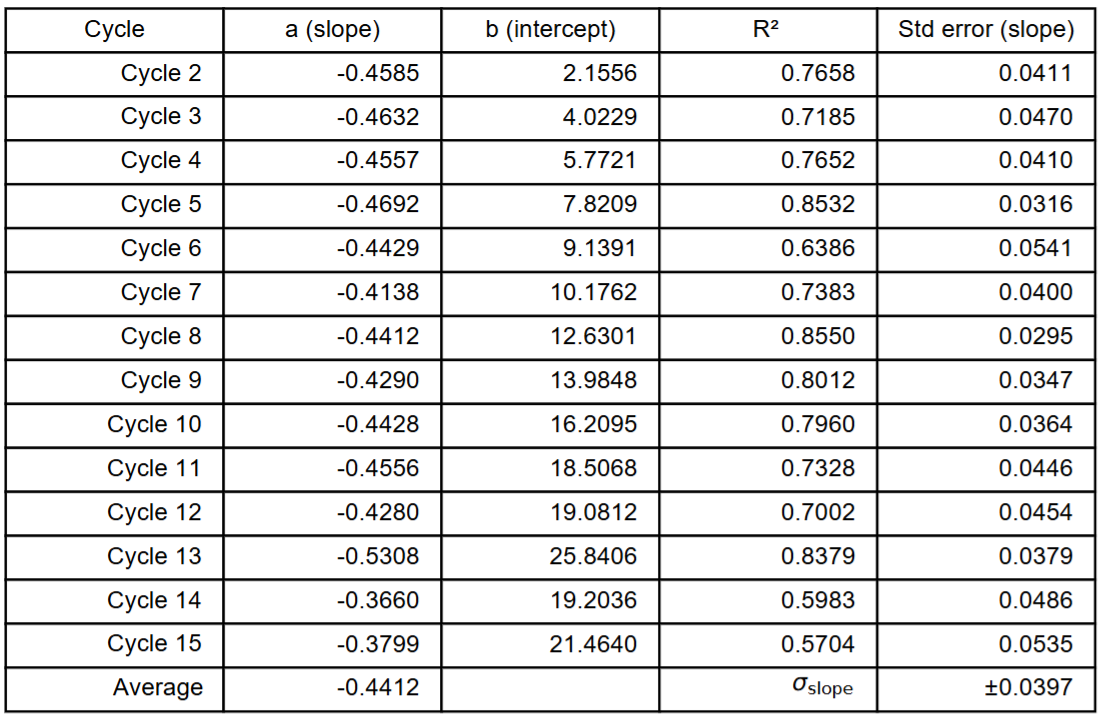
**

**
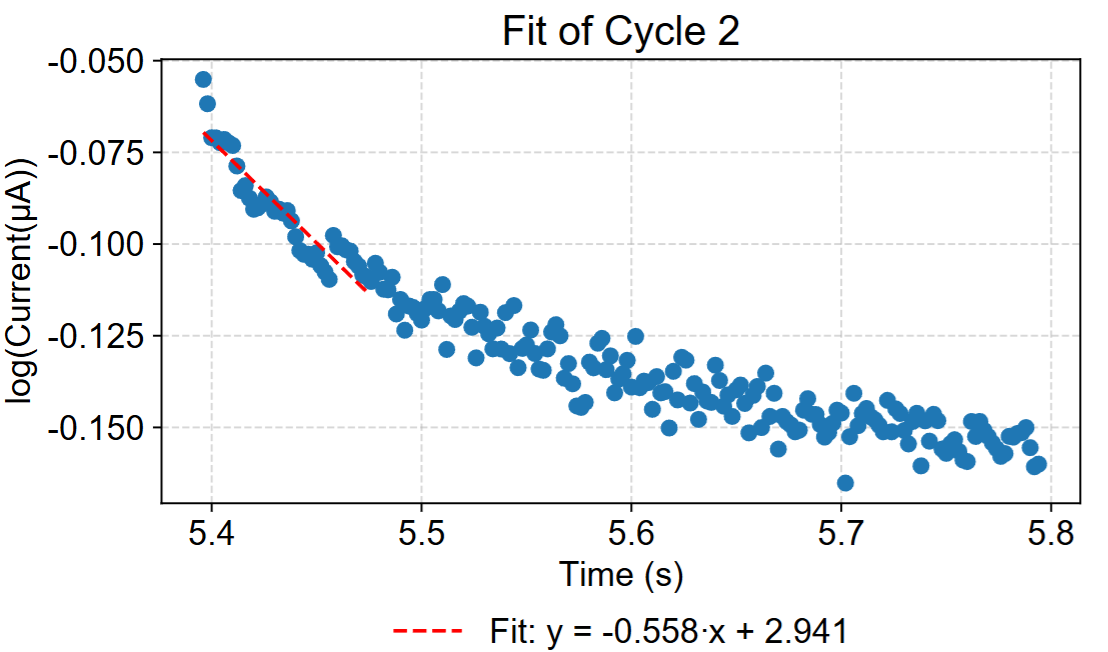
**

**Figure S24.** Fitting of the non-diffusion-limited region of the chronoamperometry data for BNAH oxidation in the presence of 3-chloropyridine with laser power attenuated by 50%.

**Table S2.** Fitting parameters of the non-diffusion-limited region of the chronoamperometry data for BNAH oxidation in the presence of 3-chloropyridine with laser power attenuated by 50%.


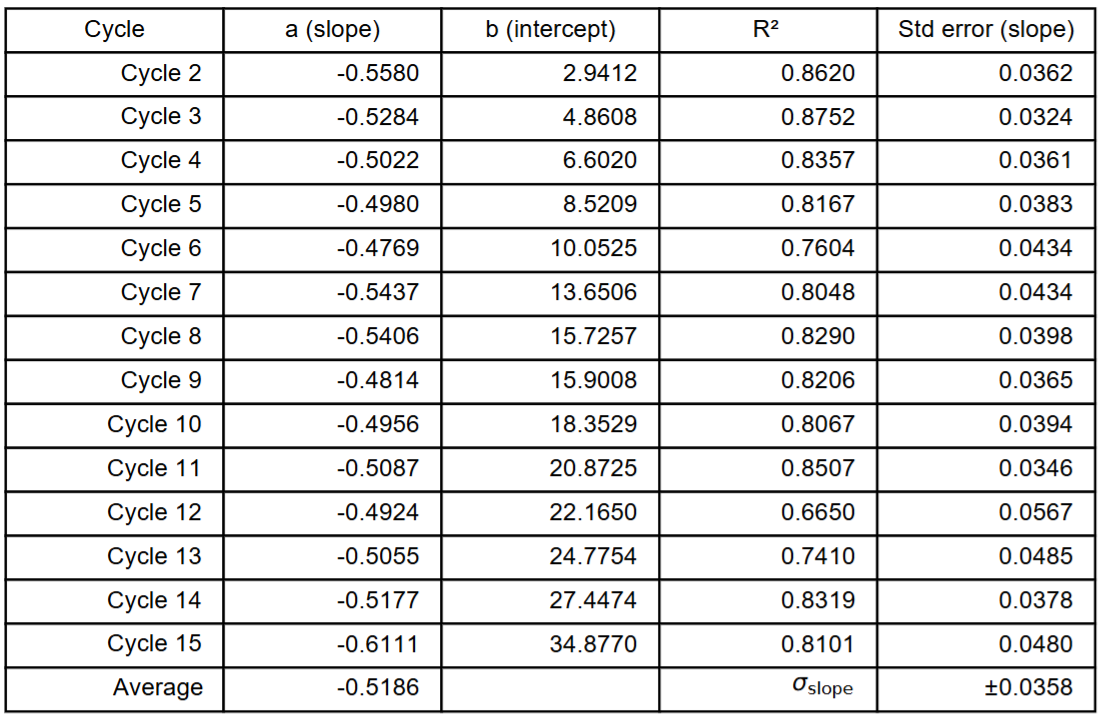


**
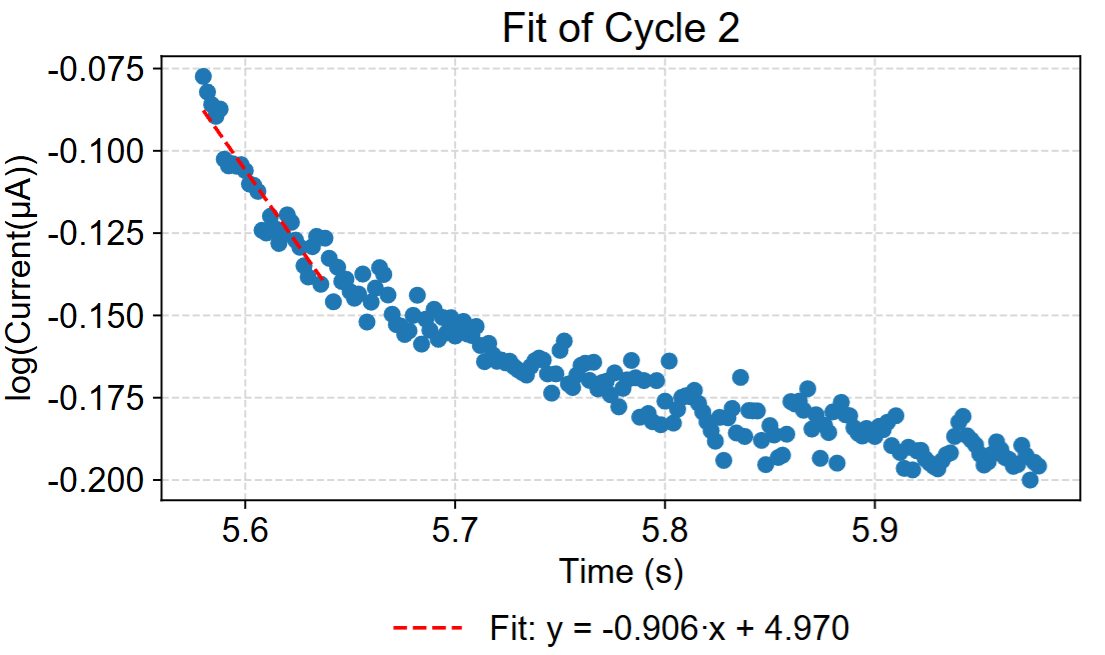
**

**Figure S25.** Fitting of the non-diffusion-limited region of the chronoamperometry data for BNAH oxidation in the presence of 3-acetylpyridine with laser power attenuated by 50%.

**Table S3.** Fitting parameters of the non-diffusion-limited region of the chronoamperometry data for BNAH oxidation in the presence of 3-acetylpyridine with laser power attenuated by 50%.


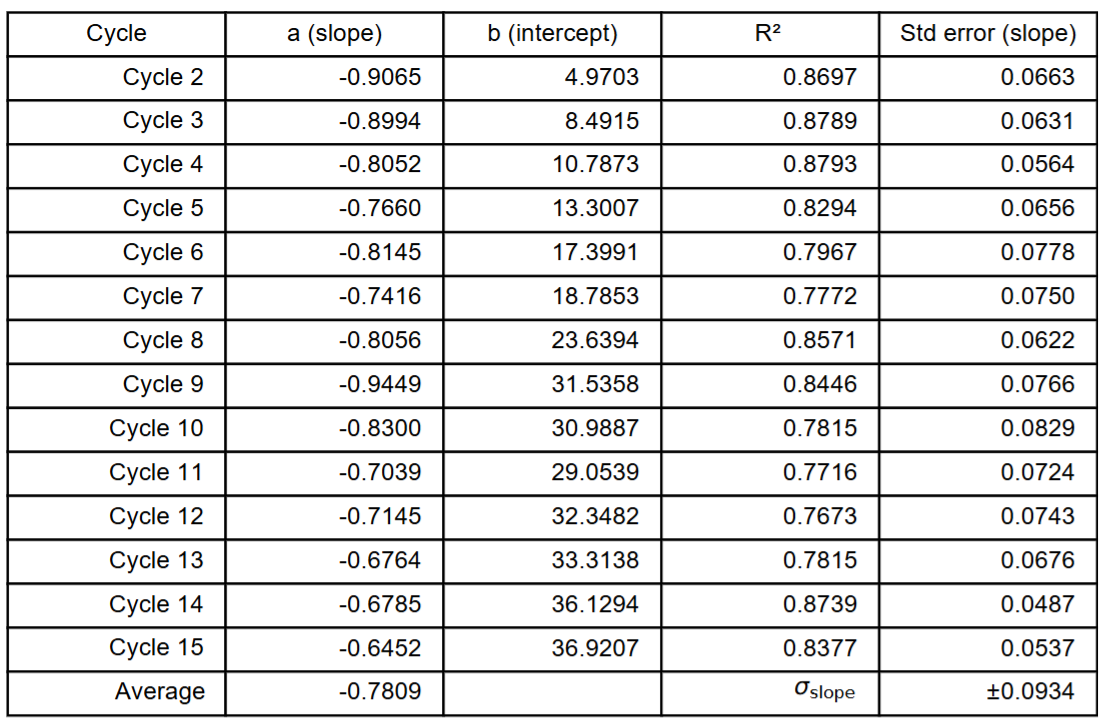


**
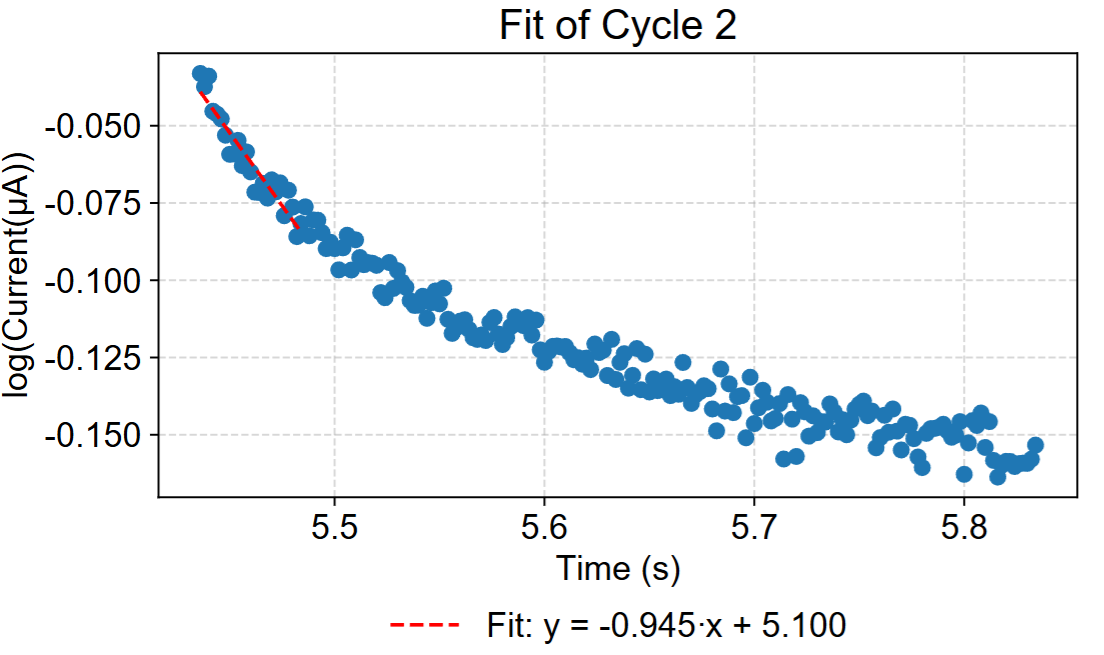
**

**Figure S26.** Fitting of the non-diffusion-limited region of the chronoamperometry data for BNAH oxidation in the presence of pyridine with laser power attenuated by 50%.

**Table S4.** Fitting parameters of the non-diffusion-limited region of the chronoamperometry data for BNAH oxidation in the presence of pyridine with laser power attenuated by 50%.


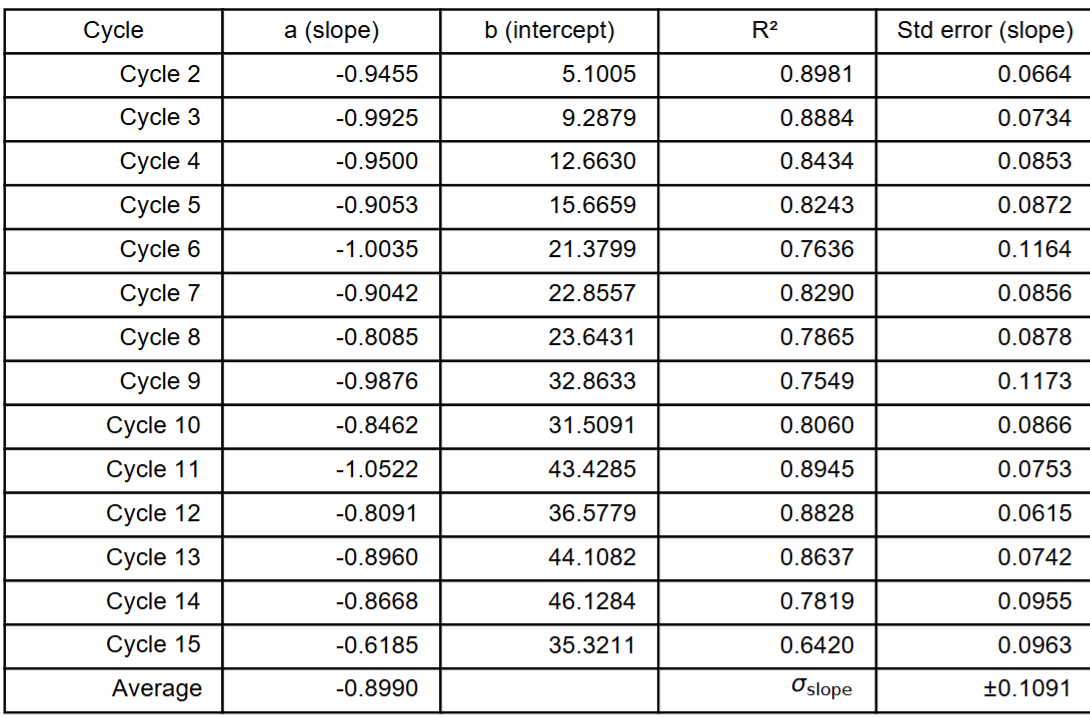


**
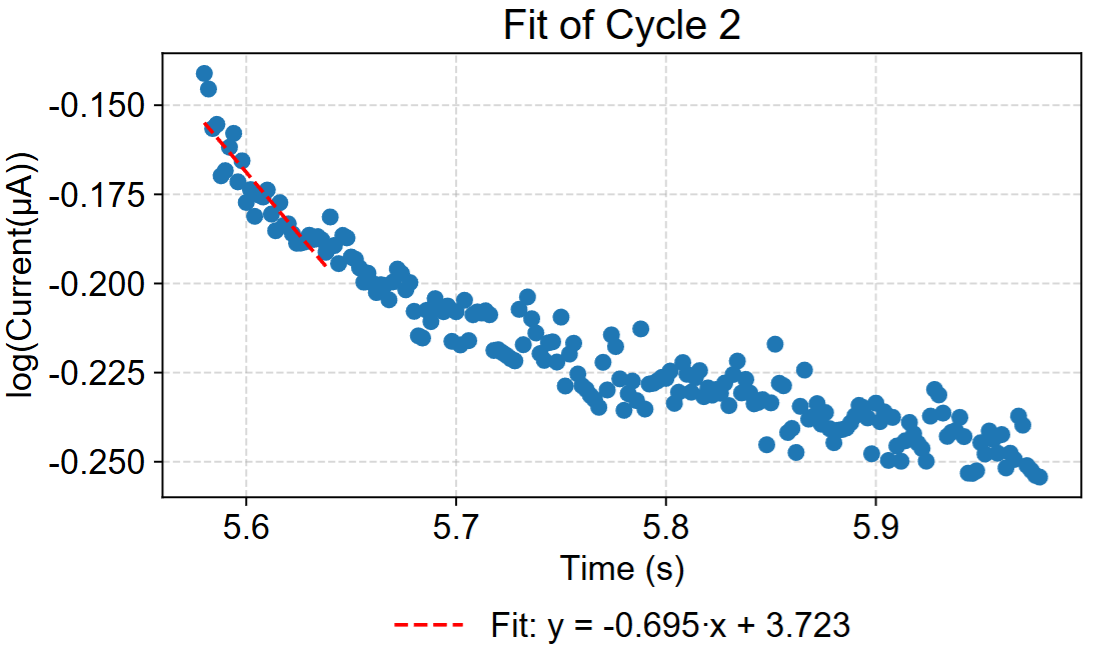
**

**Figure S27.** Fitting of the non-diffusion-limited region of the chronoamperometry data for BNAH oxidation in the presence of 4-methoxypyridine with laser power attenuated by 50%.

**Table S5.** Fitting parameters of the non-diffusion-limited region of the chronoamperometry data for BNAH oxidation in the presence of 4-methoxypyridine with laser power attenuated by 50%.


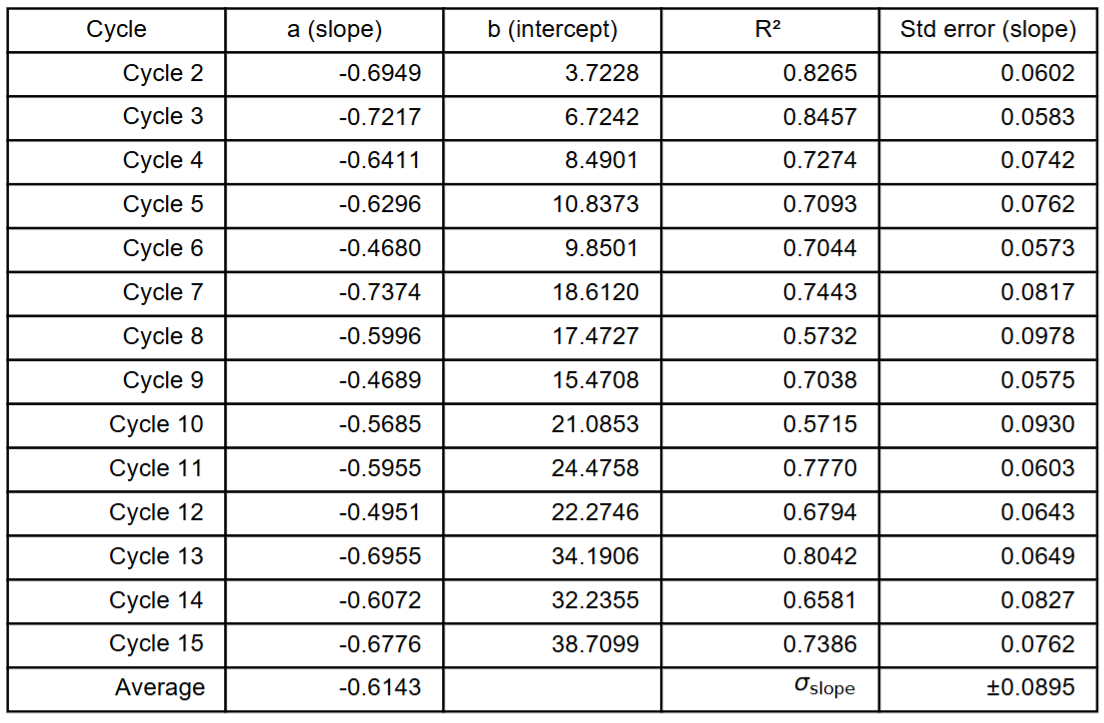


**
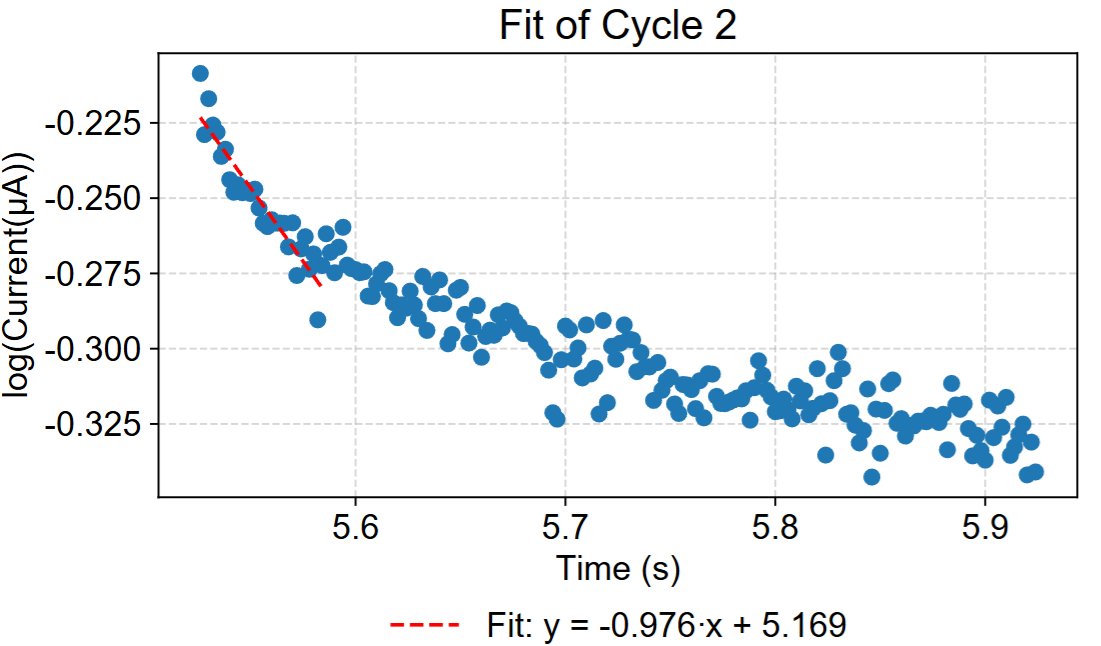
**

**Figure S28.** Fitting of the non-diffusion-limited region of the chronoamperometry data for BNAH oxidation in the presence of 4-aminopyridine with laser power attenuated by 50%.

**Table S6.** Fitting parameters of the non-diffusion-limited region of the chronoamperometry data for BNAH oxidation in the presence of 4-aminopyridine with laser power attenuated by 50%.

**
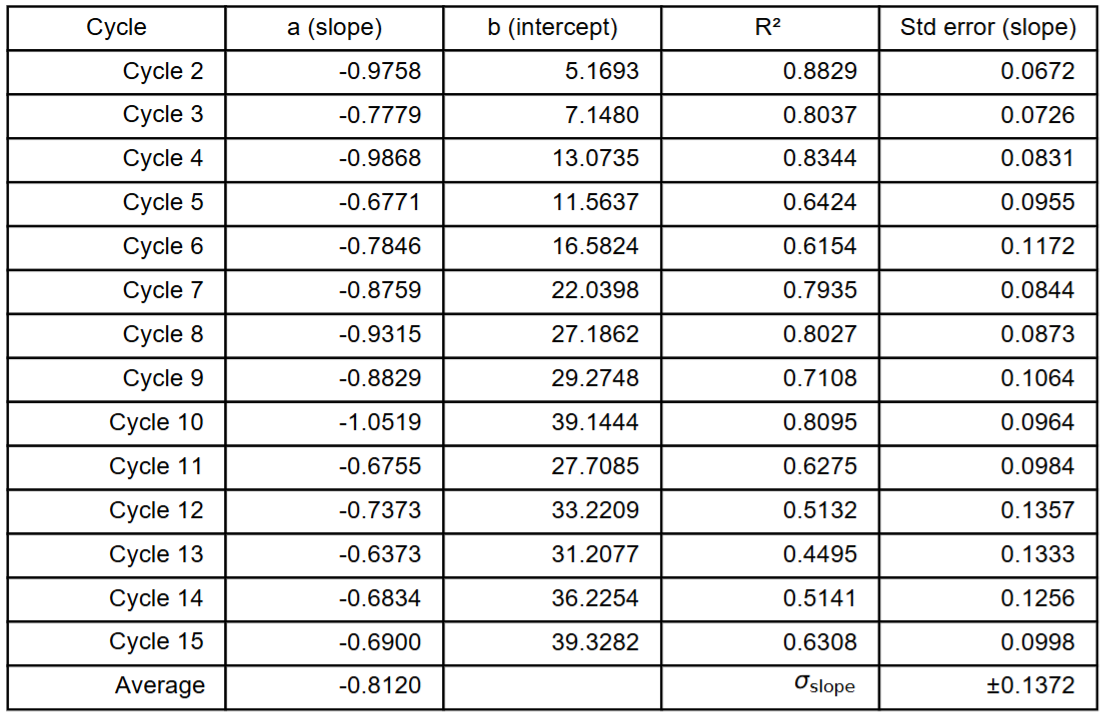
**

**
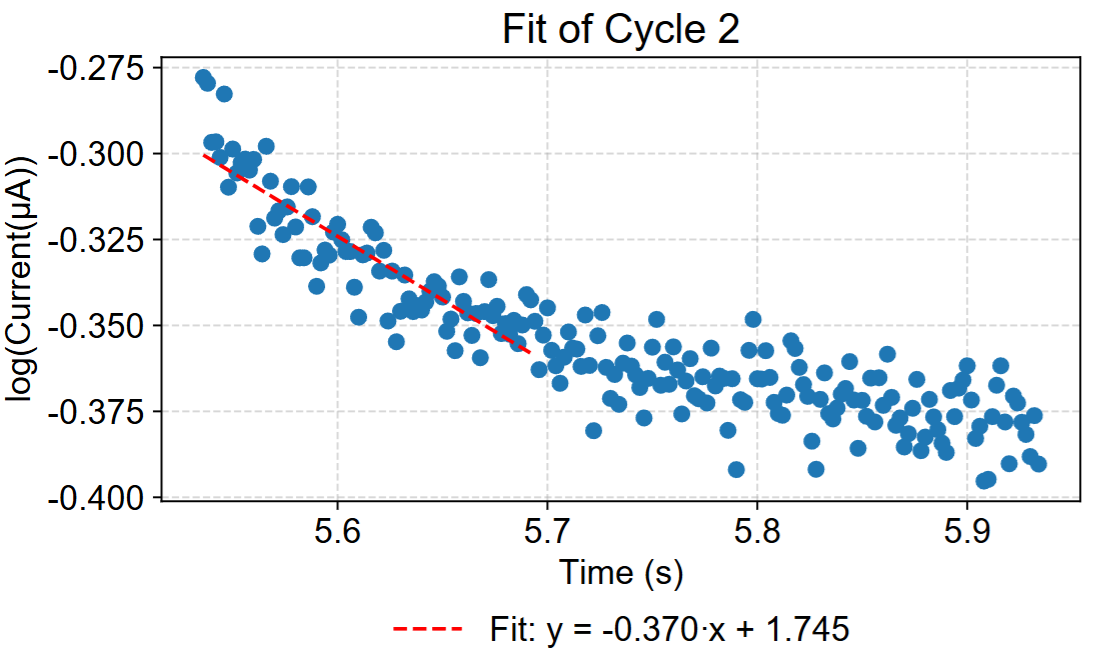
**

**Figure S29.** Fitting of the non-diffusion-limited region of the chronoamperometry data for BNAH oxidation in the presence of piperidine with laser power attenuated by 50%.

**Table S7.** Fitting parameters of the non-diffusion-limited region of the chronoamperometry data for BNAH oxidation in the presence of piperidine with laser power attenuated by 50%.

**
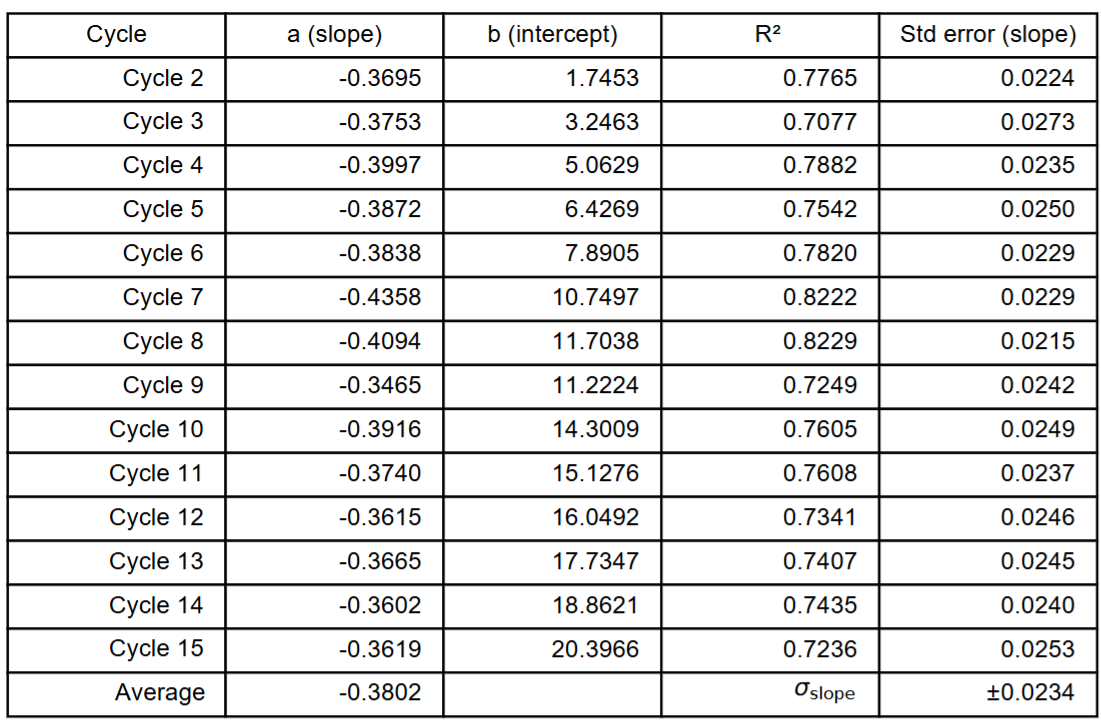
**

**
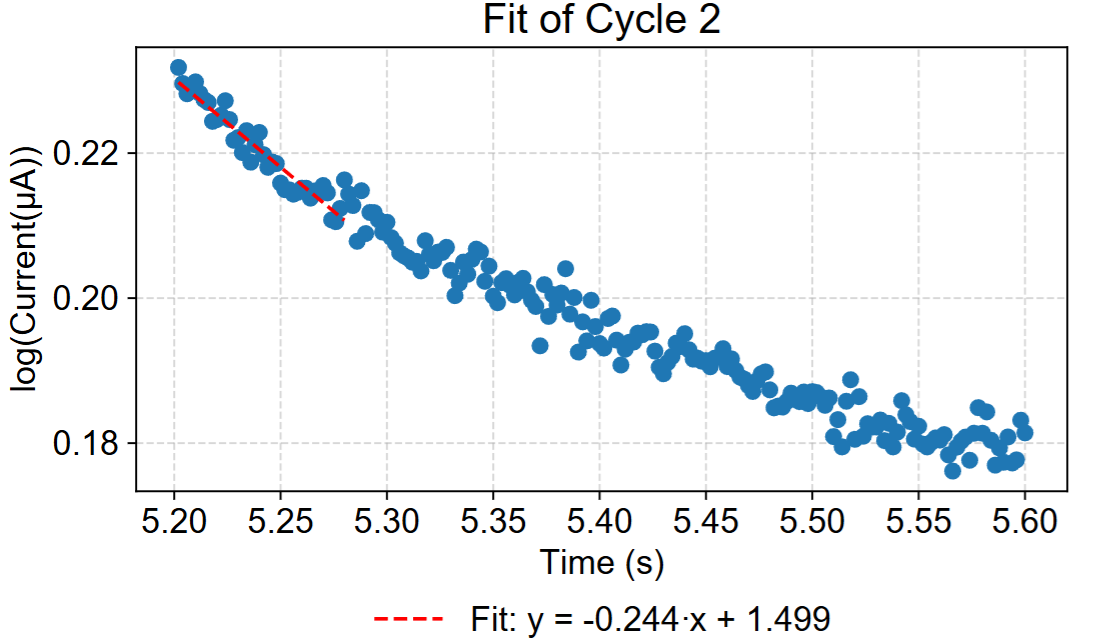
**

**Figure S30.** Fitting of the non-diffusion-limited region of the chronoamperometry data for BNAH oxidation in the presence of 2,6-dimethoxypyridine, with the laser power attenuated by 60%.

**Table S8.** Fitting parameters of the non-diffusion-limited region of the chronoamperometry data for BNAH oxidation in the presence of 2,6-dimethoxypyridine, with the laser power attenuated by 60%.


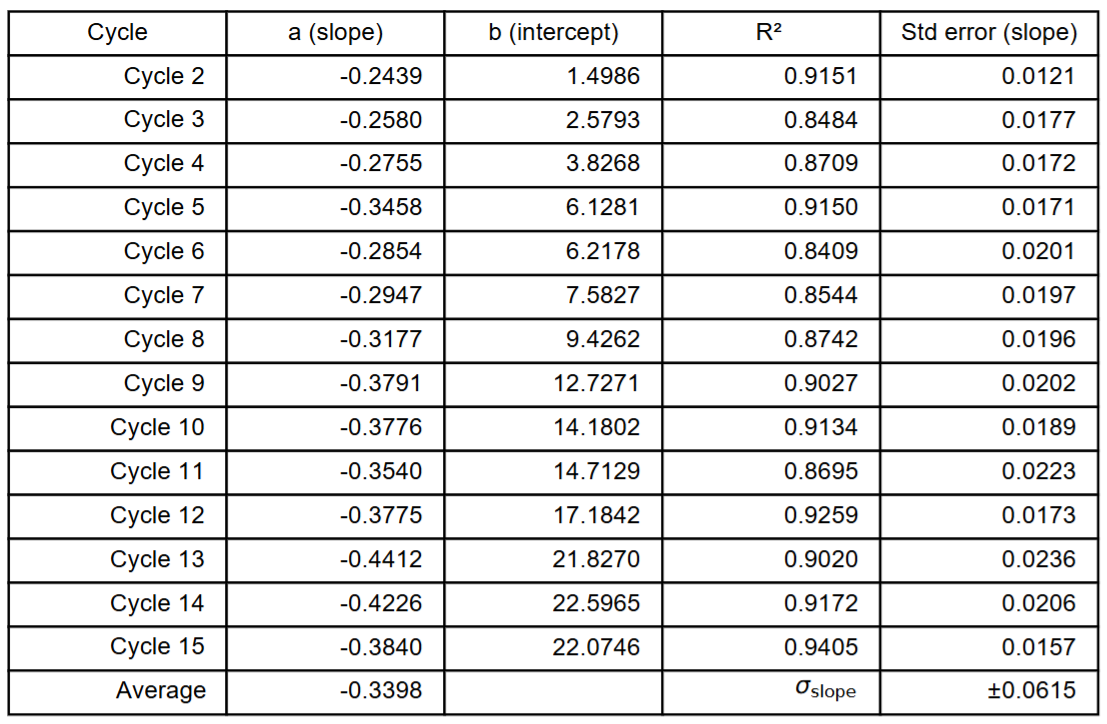


**
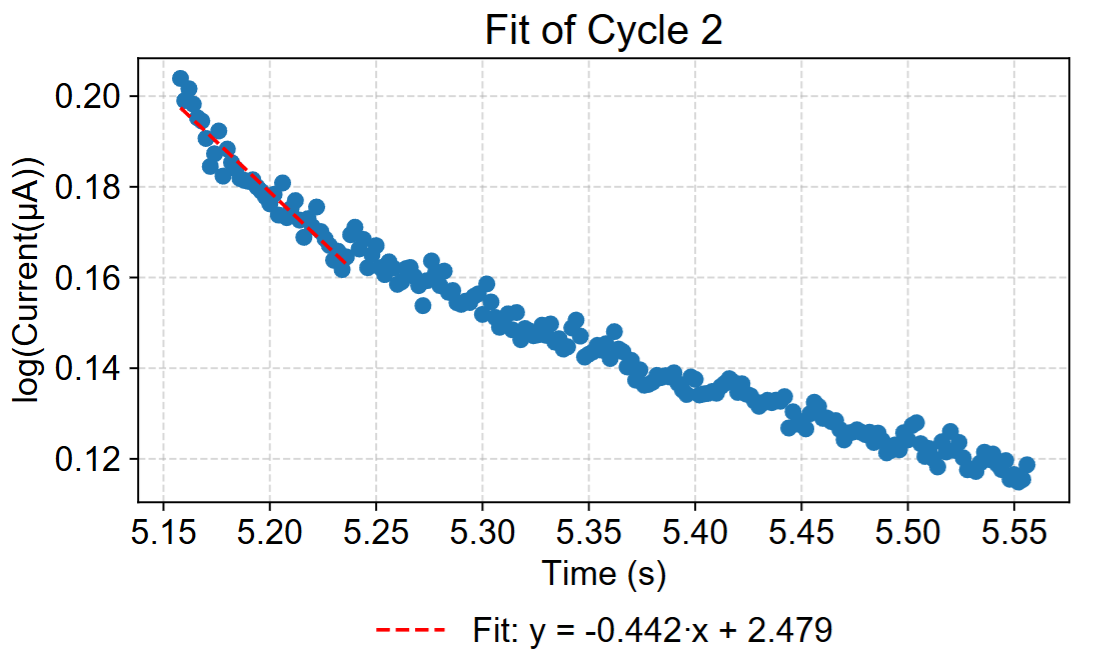
**

**Figure S31.** Fitting of the non-diffusion-limited region of the chronoamperometry data for BNAH oxidation in the presence of 3-chloropyridine with laser power attenuated by 60%.

**Table S9.** Fitting parameters of the non-diffusion-limited region of the chronoamperometry data for BNAH oxidation in the presence of 3-chloropyridine with laser power attenuated by 60%.


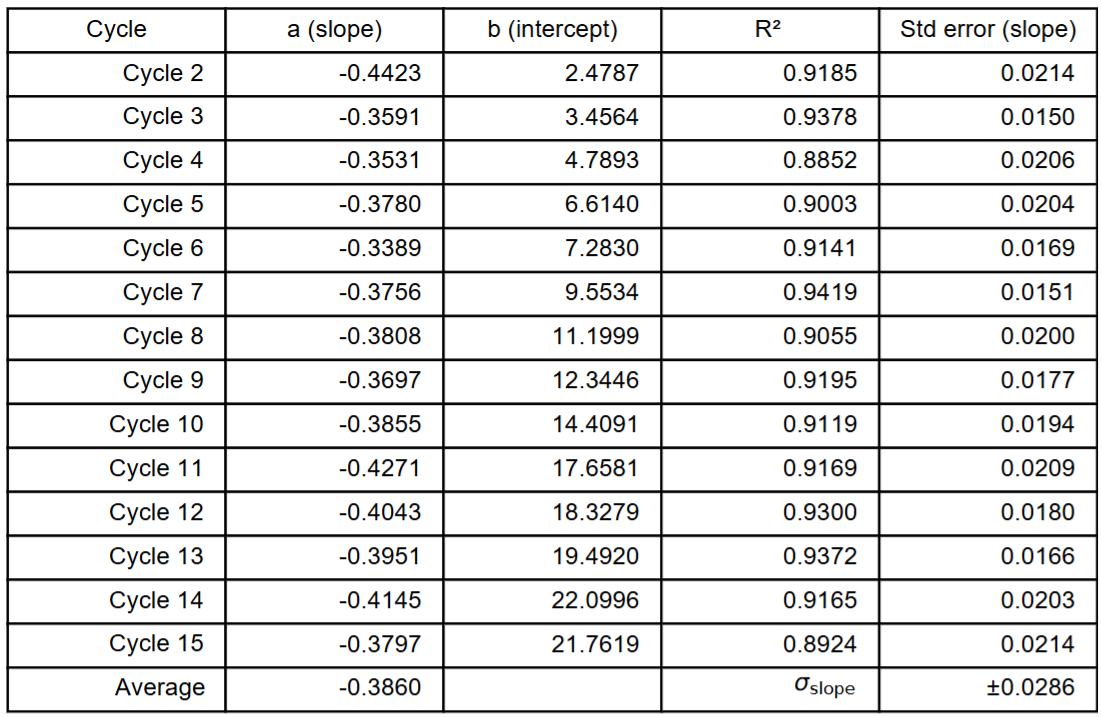


**
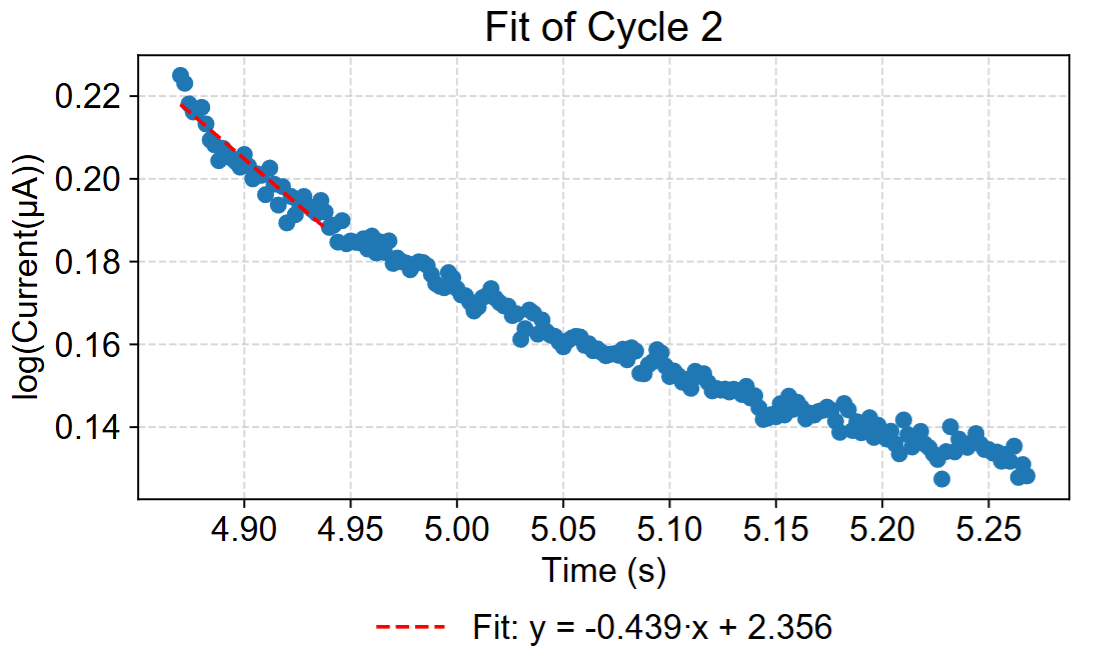
**

**Figure S32.** Fitting of the non-diffusion-limited region of the chronoamperometry data for BNAH oxidation in the presence of 3-acetylpyridine with laser power attenuated by 60%.

**Table S10.** Fitting parameters of the non-diffusion-limited region of the chronoamperometry data for BNAH oxidation in the presence of 3-acetylpyridine with laser power attenuated by 60%.


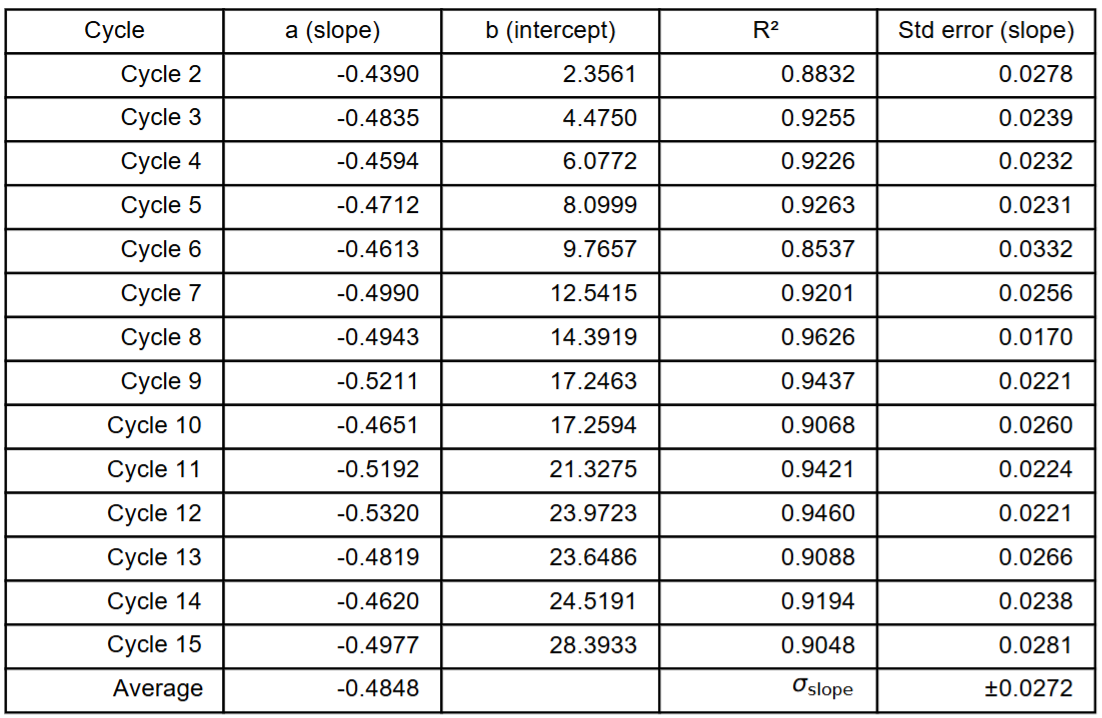


**
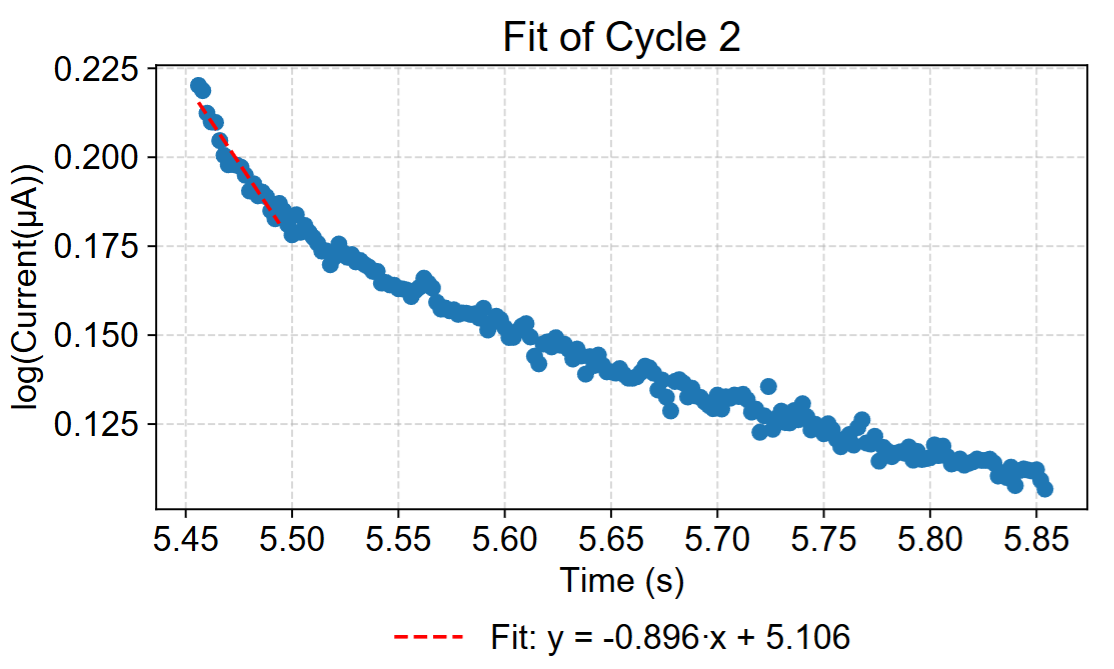
**

**Figure S33.** Fitting of the non-diffusion-limited region of the chronoamperometry data for BNAH oxidation in the presence of pyridine with laser power attenuated by 60%.

**Table S11.** Fitting parameters of the non-diffusion-limited region of the chronoamperometry data for BNAH oxidation in the presence of pyridine with laser power attenuated by 60%.


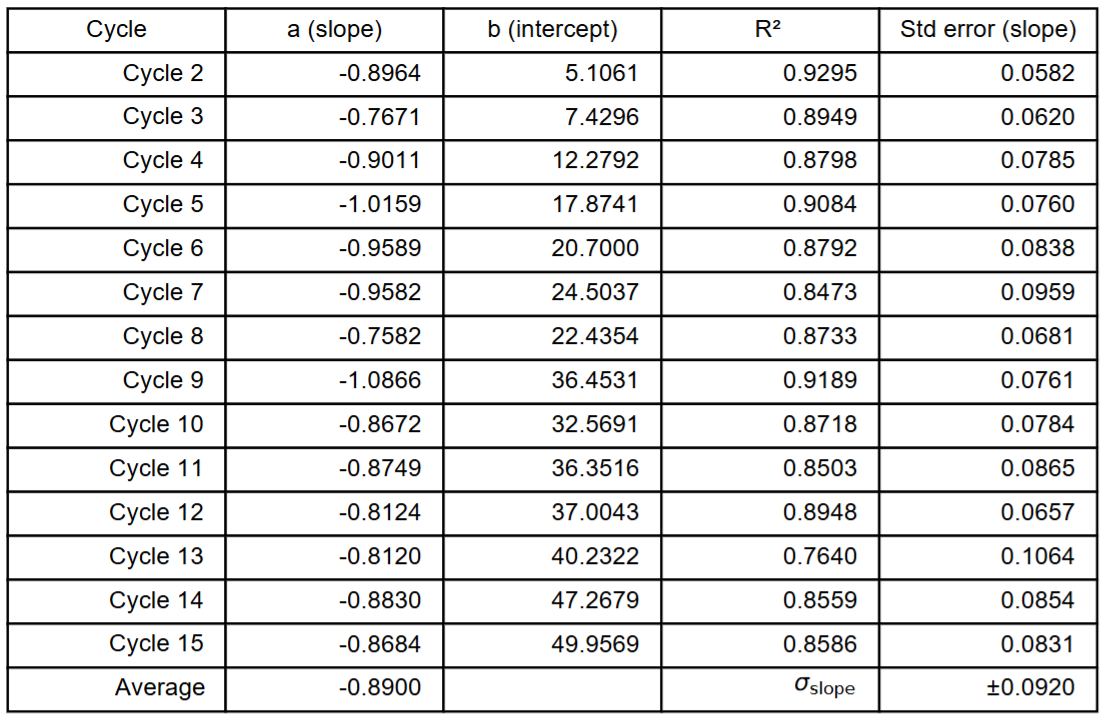


**
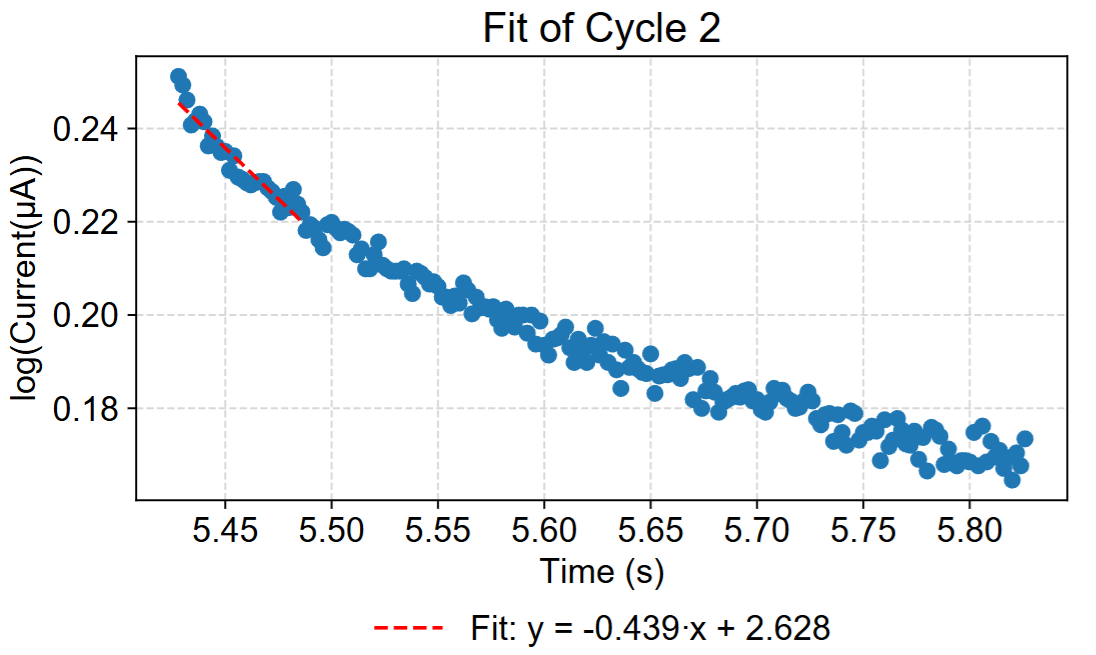
**

**Figure S34.** Fitting of the non-diffusion-limited region of the chronoamperometry data for BNAH oxidation in the presence of 4-methoxypyridine with laser power attenuated by 60%.

**Table S12.** Fitting parameters of the non-diffusion-limited region of the chronoamperometry data for BNAH oxidation in the presence of 4-methoxypyridine with laser power attenuated by 60%.


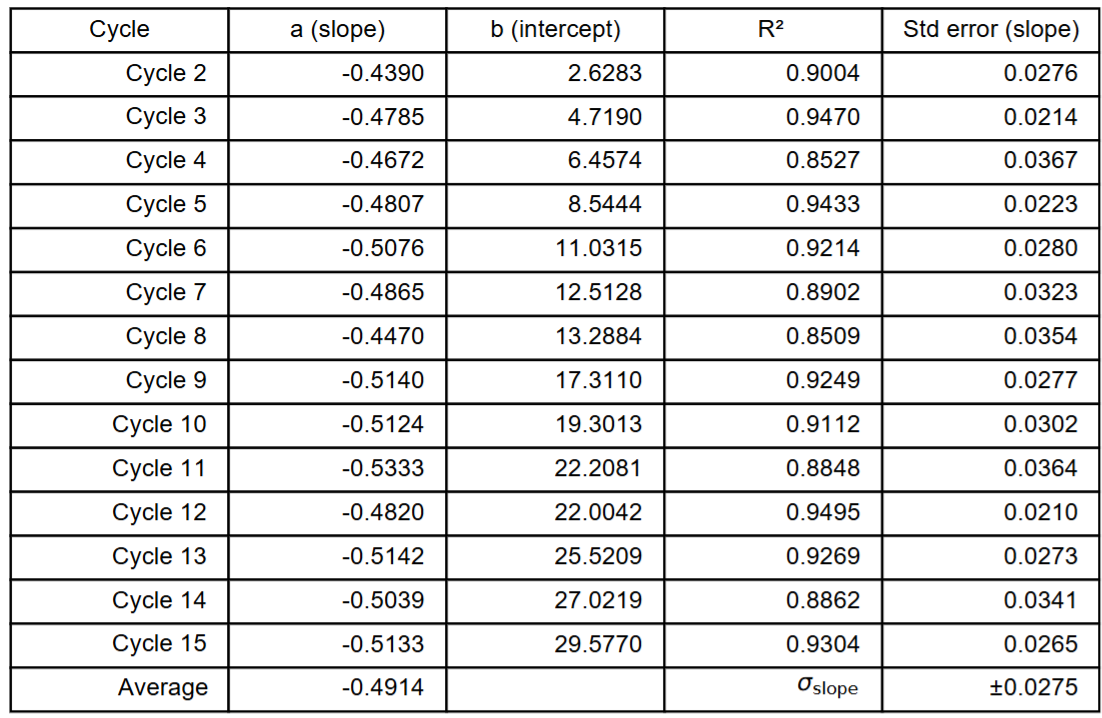


**
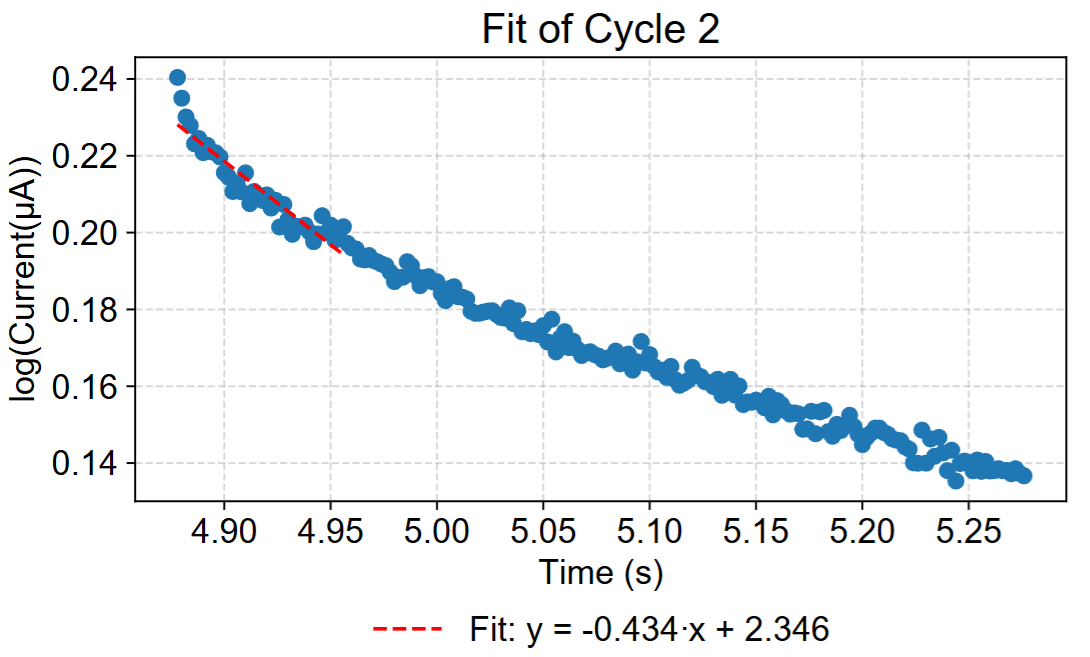
**

**Figure S35.** Fitting of the non-diffusion-limited region of the chronoamperometry data for BNAH oxidation in the presence of 4-aminopyridine with laser power attenuated by 60%.

**Table S13.** Fitting parameters of the non-diffusion-limited region of the chronoamperometry data for BNAH oxidation in the presence of 4-aminopyridine with laser power attenuated by 60%.

**
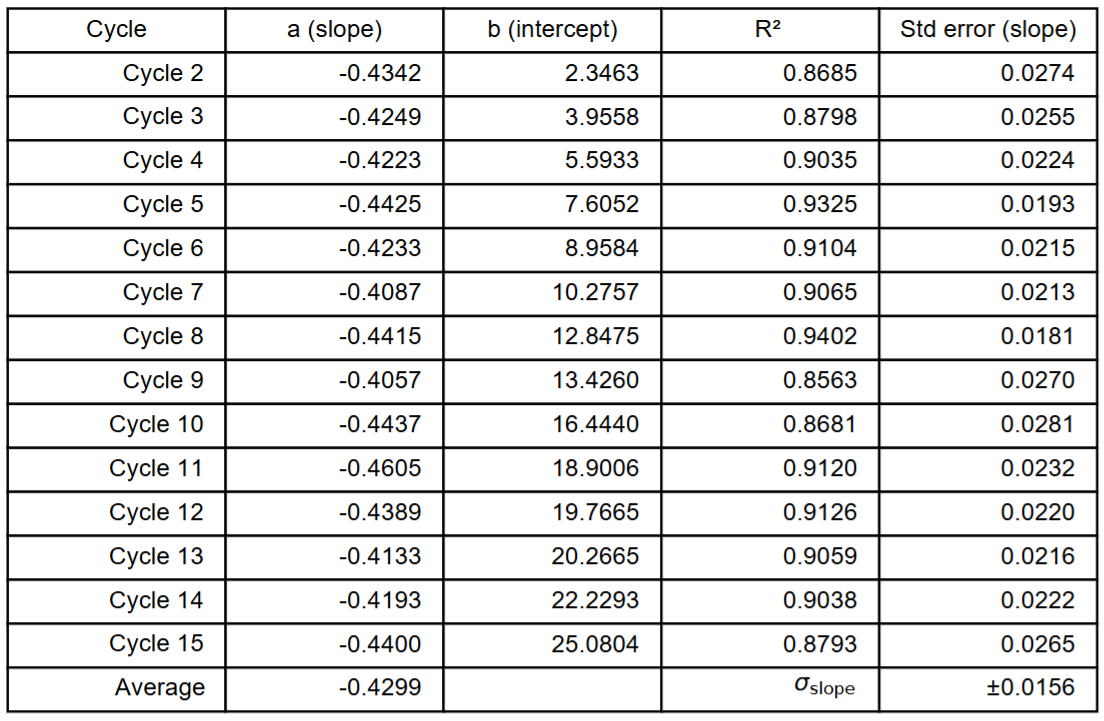
**

**
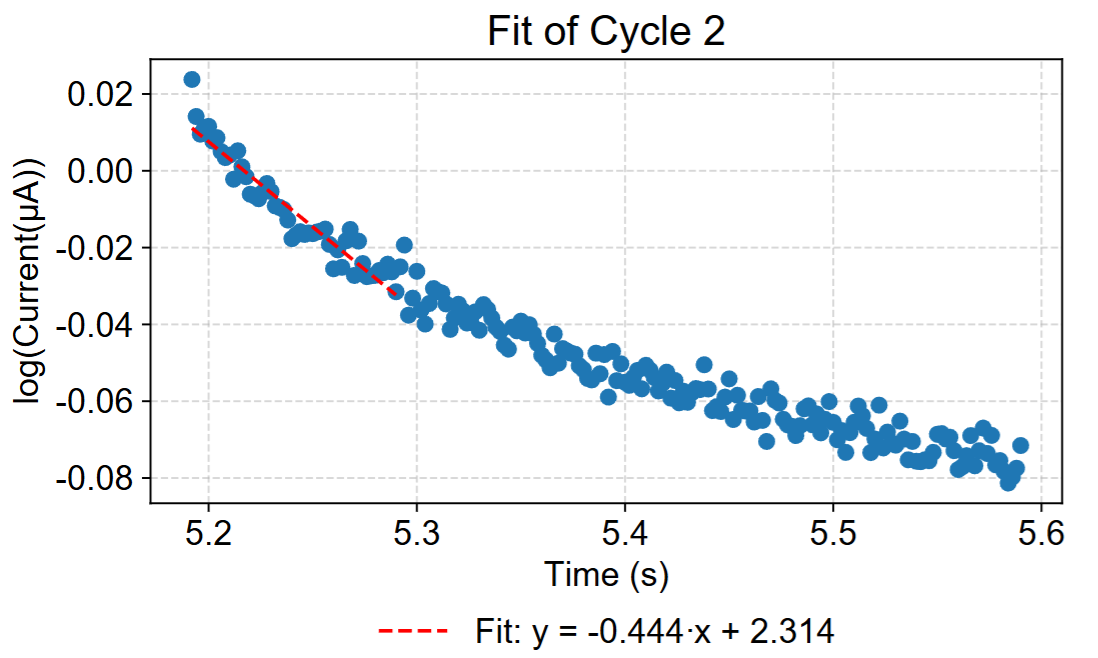
**

**Figure S36.** Fitting of the non-diffusion-limited region of the chronoamperometry data for BNAH oxidation in the presence of piperidine with laser power attenuated by 60%.

**Table S14.** Fitting parameters of the non-diffusion-limited region of the chronoamperometry data for BNAH oxidation in the presence of piperidine with laser power attenuated by 60%.


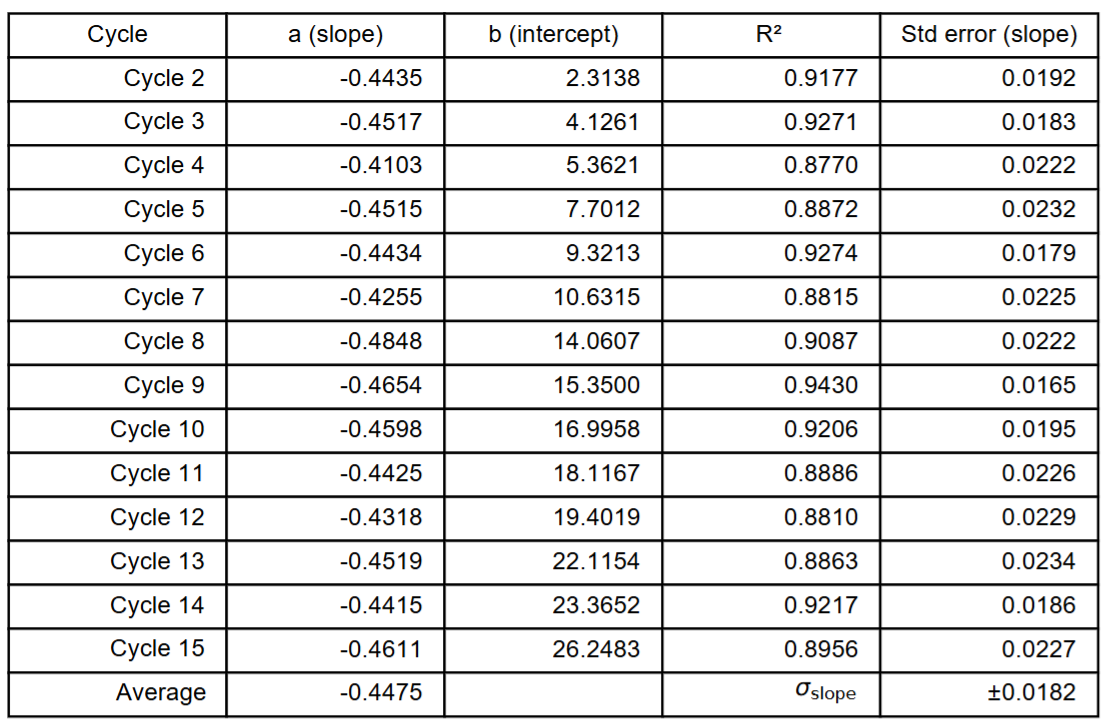


**References:**

1. [] P. Sekar, R. Bericat-Vadell, Y. Patehebieke, P. Bröqvist, C.-J. Wallentin, M. Görlin, J. Sá, Decoupling plasmonic hot carrier from thermal catalysis via electrode engineering. *Nano Lett.* **2024**, *24*, 8619-8625. [↑](#endnote-ref-1)
